# Contractile force assessment methods for in vitro skeletal muscle tissues

**DOI:** 10.7554/eLife.77204

**Published:** 2022-05-23

**Authors:** Camila Vesga-Castro, Javier Aldazabal, Ainara Vallejo-Illarramendi, Jacobo Paredes

**Affiliations:** 1 University of Navarra, Tecnun School of Engineering, Manuel de Lardizábal San Sebastian Spain; 2 University of Navarra, Biomedical Engineering Center, Campus Universitario Pamplona Spain; 3 Group of Neurosciences, Department of Pediatrics, UPV/EHU, Hospital Donostia - IIS Biodonostia San Sebastian Spain; 4 CIBERNED, Instituto de Salud Carlos III, Ministry of Science, Innovation, and Universities Madrid Spain; University of Cambridge United Kingdom; Icahn School of Medicine at Mount Sinai United States

**Keywords:** contractile force, skeletal muscle, tissue engineering, stimulation

## Abstract

Over the last few years, there has been growing interest in measuring the contractile force (CF) of engineered muscle tissues to evaluate their functionality. However, there are still no standards available for selecting the most suitable experimental platform, measuring system, culture protocol, or stimulation patterns. Consequently, the high variability of published data hinders any comparison between different studies. We have identified that cantilever deflection, post deflection, and force transducers are the most commonly used configurations for CF assessment in 2D and 3D models. Additionally, we have discussed the most relevant emerging technologies that would greatly complement CF evaluation with intracellular and localized analysis. This review provides a comprehensive analysis of the most significant advances in CF evaluation and its critical parameters. In order to compare contractile performance across experimental platforms, we have used the specific force (sF, kN/m^2^), CF normalized to the calculated cross-sectional area (CSA). However, this parameter presents a high variability throughout the different studies, which indicates the need to identify additional parameters and complementary analysis suitable for proper comparison. We propose that future contractility studies in skeletal muscle constructs report detailed information about construct size, contractile area, maturity level, sarcomere length, and, ideally, the tetanus-to-twitch ratio. These studies will hopefully shed light on the relative impact of these variables on muscle force performance of engineered muscle constructs. Prospective advances in muscle tissue engineering, particularly in muscle disease models, will require a joint effort to develop standardized methodologies for assessing CF of engineered muscle tissues.

## Introduction

The main function of skeletal muscle is to produce contractile force (CF) (Vandenburgh et al., 2008; Shadrin et al., 2016), which is necessary for locomotion, respiration, and metabolic processes (Kaji et al., 2010; Qazi et al., 2015; Wang et al., 2019; Yusuf and Brand-Saberi, 2012). CF may be compromised during aging or due to major injuries or genetic mutations, such as in muscular dystrophies. Calcium (Ca^2+^) homeostasis, which is usually dysregulated in these conditions, has a profound impact on force production (Vallejo-Illarramendi et al., 2014; Al Tanoury et al., 2021). Muscular contraction is initiated by motoneurons, which release acetylcholine (ACh) upon activation by action potentials. ACh causes sarcolemmal depolarization and opening of calcium channels at the sarcolemma and the sarcoplasmic reticulum. Ca^2+^ diffuses throughout the sarcomere and initiates contraction by a cross-bridge cycle between actin and myosin (Widmaier and Raff, 2008; Fernández-Costa et al., 2021; Santoso et al., 2021).

For years, standard 2D cellular cultures and animal models have been used to understand muscle physiopathology and to develop novel treatments for muscle diseases. Despite the many discoveries regarding those models, a comparison with native human muscle suggests that major challenges remain in order to obtain a fully representative or trustworthy model (Horvath et al., 2016; Santoso et al., 2021; Wang et al., 2019). Characteristics such as size, drug diffusion, or immune response are difficult to match with the ones occurring in the human body (Fernández-Costa et al., 2021; Khodabukus, 2021). For instance, the muscular dystrophy X-linked mouse (mdx) is a well-accepted preclinical model for Duchenne Muscular Dystrophy (DMD) (Bersini et al., 2018; Lasa-Fernandez et al., 2020; Al Tanoury et al., 2021). However, mdx mice present substantial differences compared to Duchenne patients regarding muscle regeneration, Ca^2+^ handling, and life expectancy (Raymackers et al., 2003; Toral-Ojeda et al., 2018; Yoshioka et al., 2021b). Other DMD animal models (dogs or pigs) seem to better recapitulate the human disease, but they entail substantial drawbacks or challenges such as higher variability in disease progression, longer experimental times, and, obviously, much higher costs (Smith et al., 2016; Kornegay, 2017).

**Figure 1.**
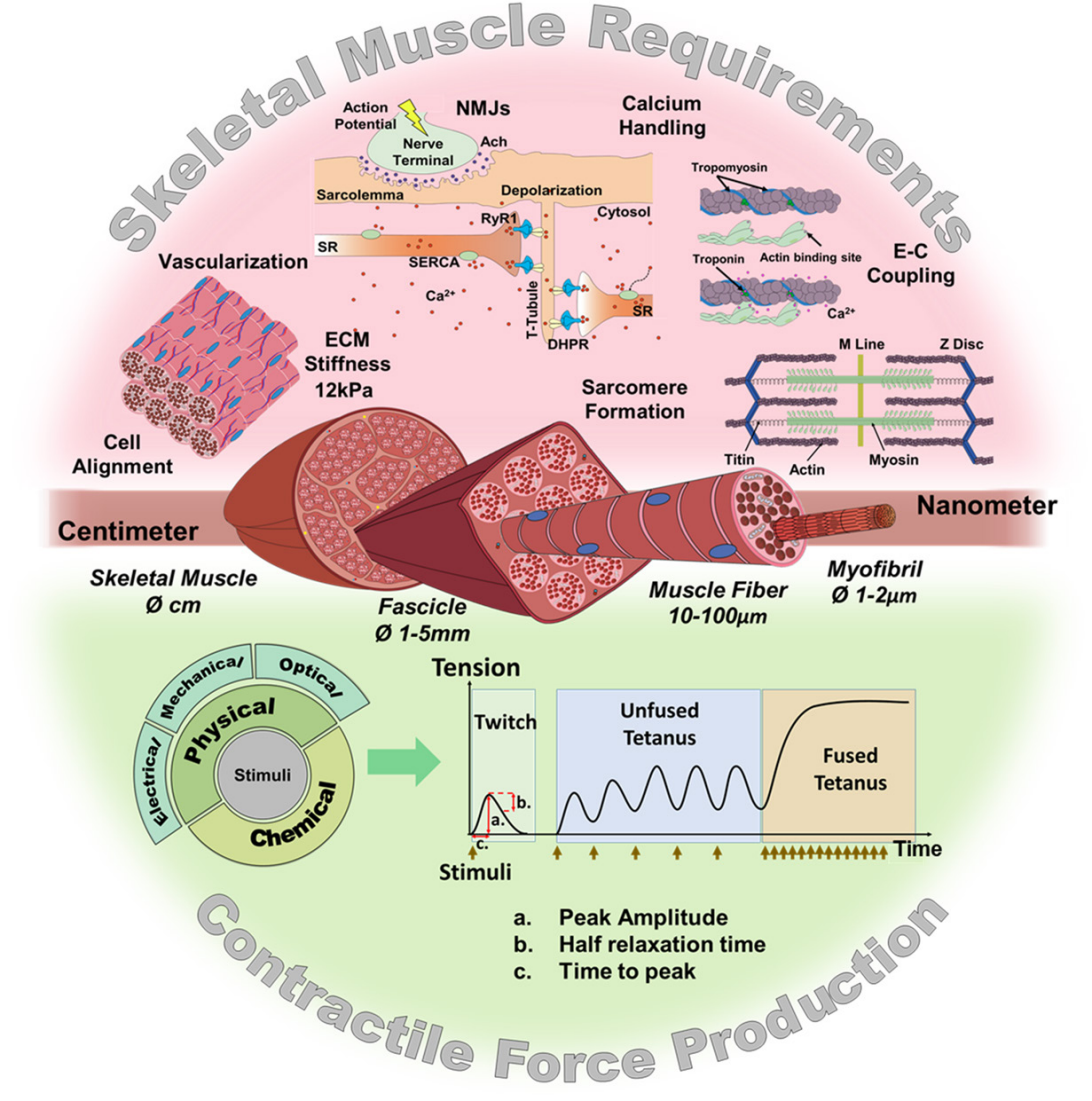
Skeletal muscle structure and requirements for contractile force production. (Top) Summary of the main requirements to enable contractility in skeletal muscle in vitro models. (Middle) Representation of the muscular hierarchy and (bottom) summary of contractile stimuli and contraction profiles.

In vitro models, as an alternative to animal models, are already providing promising results in different applications such as disease modeling (Shimizu et al., 2017; Nesmith et al., 2016; Badu-Mensah et al., 2020; Fernández-Garibay et al., 2021), drug discovery (Vandenburgh, 2010; Ikeda et al., 2017; Afshar et al., 2020; Alave Reyes-Furrer et al., 2021), gene therapy (Powell et al., 1999; Vandenburgh et al., 1996), reconstructive surgery (Brady et al., 2008; Kim et al., 2018; Gilbert-Honick et al., 2020), or even as bio-actuators in biohybrid systems (Yamamoto et al., 2011; Cvetkovic et al., 2014; Gao et al., 2021). Indeed, the latest technological advances in tissue engineering such as 3D bioprinting or the development of novel biomaterials and culture platforms (Capel et al., 2019; Denes et al., 2019; Costantini et al., 2021; Gupta et al., 2021; Peper et al., 2021) are facilitating the development of better in vitro skeletal muscle constructs. To this end, researchers have recapitulated many native skeletal muscle features, including contraction (Madden et al., 2015; Urciuolo et al., 2020; Akiyama et al., 2021; Alave Reyes-Furrer et al., 2021), vascularization (van der Schaft et al., 2011; Bersini et al., 2018; Osaki et al., 2018a; Gilbert-Honick et al., 2018), mechanical tension (Okano and Matsuda, 1998; Vandenburgh and Karlisch, 1989), neuromuscular junction (NMJ) (Afshar Bakooshli et al., 2019; Yoshioka et al., 2020; Vila et al., 2021), and muscle stiffness (Urciuolo et al., 2020). These features, represented in Figure 1 (top), are considered key requirements for generating functional and mature skeletal muscle constructs.

The current gold standard for the characterization of in vitro muscle models includes expression analysis of myogenic markers, determination of the fusion index, and evaluation of striation pattern and ACh receptor aggregation (Bajaj et al., 2011; Toral-Ojeda et al., 2016; Toral-Ojeda et al., 2018). Whereas this information is helpful for understanding the basic differentiation status of the culture, CF provides further physiological information about the degree of tissue maturation and functionality (Madden et al., 2015; Ikeda et al., 2016; Juhas et al., 2016; Nagashima et al., 2020). Indeed, CF evaluation has already established its usefulness as a primary outcome measure in drug screening platforms (Ikeda et al., 2017; Shimizu et al., 2017; Vandenburgh et al., 2009), analyzing the performance of different cellular sources (Nagashima et al., 2020; Shimizu et al., 2020), assessing the effect of scaffold composition (Boontheekul et al., 2007; Hinds et al., 2011), or even evaluating the influence of heat stress (Takagi et al., 2018), and media supplements on muscle development (Martin et al., 2017; Mills et al., 2019; Xu et al., 2019).

However, it is quite challenging to compare CF magnitudes obtained under different experimental conditions in which crucial key factors differ such as culture platforms (Anene-Nzelu et al., 2013; Khodabukus and Baar, 2016), culturing methods, cell lines, scaffold materials (Zhuang et al., 2020; Gilbert-Honick et al., 2020; Perez-Puyana et al., 2021) or even construct size. Consequently, there is a wide range of results from different research laboratories worldwide that are difficult to compare or interpret. This fact raises the need to define homogeneous units and to standardize the methodology for CF assessment in order to facilitate proper comparison among studies.

## Contractile force induction

Several stimulation mechanisms may be used to trigger contraction of in vitro muscle constructs at specific paces: CF may be induced by chemical (Osaki et al., 2018b; Afshar et al., 2020; Yoshioka et al., 2020; Hofemeier et al., 2021), or physical stimuli (Figure 1, bottom). As for physical stimuli, they comprise electrical (Juhas and Bursac, 2014; Madden et al., 2015; Santoso et al., 2021), mechanical (Kim et al., 2019), and optical stimulation (Neal et al., 2015; Mills et al., 2019; Uchimura et al., 2021; Hofemeier et al., 2021).

Electrical stimulation is the most frequently used mechanism to induce CF in engineered muscles by mimicking motoneuron activity on these tissues (Cheng et al., 2014b; Rangarajan et al., 2014; Ikeda et al., 2016; Akiyama et al., 2021). It has also been used during muscle development to improve muscle size, sarcomere formation, Ca^2+^ transients, and CF production (Park et al., 2008; Langelaan et al., 2011; Khodabukus et al., 2019). Electrical stimulation causes depolarization of the muscle cell membrane, which triggers excitation-contraction coupling (Merrill et al., 2005; Uchimura et al., 2021). It is essential to optimize the electrical parameters for each experimental setup and culture stage, since inadequate stimulation can result in culture damage or untoward responses, including fatigue, electrochemical damage, or electroporation effect, leading to increased membrane permeability and impaired cell function (Nikolić et al., 2017; Khodabukus and Baar, 2012; Mills et al., 2019; Ruzgys et al., 2019; Nagamine et al., 2011; Khodabukus, 2021).

Optical stimulation or photo-stimulation entails the incorporation of light-sensitive proteins such as Channelrhodopsin-2 (ChR2) by gene delivery. ChR2 is a blue-light-sensitive opsin that triggers membrane depolarization and downstream signaling cascades (Mahmoudi et al., 2017). This approach allows for an accurate spatial and temporal control of the contractile activity in a non-invasive manner, with minimal side effects to the constructs (Bruegmann et al., 2010; Asano et al., 2012; Neal et al., 2015; Vila et al., 2021). The performance of this technique depends on efficient gene delivery into the cells (Zhang et al., 2006). In particular, gene transduction with lentiviral particles has been shown to effectively generate mature 2D and 3D muscle constructs with the capability to trigger contractions optogenetically (Osaki et al., 2018b; Hofemeier et al., 2021; Cheesbrough et al., 2022).

Mechanical stimulation aims to improve muscular development by evoking the stimuli given by the skeletal system during human embryogenesis (Powell et al., 2002; Moon et al., 2008; Candiani et al., 2010). Its mechanism is based on the generation of tensile stress on the tissue construct, which triggers cellular responses mediated by mechanotransduction pathways. Depending on the parameters used, mechanical stimulation may lead to tissue enhancement and rescue of atrophy, with increased myotube number, myofiber diameter, and fusion index (Vandenburgh et al., 1991; Aguilar-Agon et al., 2019; Kim et al., 2019). However, inadequate mechanical stimulation may also induce pathological changes in muscle constructs, such as poor tissue maturation as revealed by decreased sarcomeric proteins (Akimoto et al., 2001; Boonen et al., 2010).

Finally, chemical stimulation may induce contractility of the muscle construct by direct exposure to certain biochemical compounds such as KCl, caffeine, glutamic acid, and acetylcholine (Osaki et al., 2018b; Yoshioka et al., 2020; Hofemeier et al., 2021). In addition to contraction, chemical stimulation is commonly used to induce other cellular responses, including changes in Ca^2+^ fluxes and cell size (Madden et al., 2015; Shimizu et al., 2017).

## Contractile force analysis

### Specific force

CF or tension, which is expressed in Newtons (N) is not a very informative parameter for engineered muscles, because it does not relate to the size and condition of the tissue construct. Instead, the Specific Force (sF), also referred to as mechanical stress, is the CF normalized to the cross-sectional area (CSA) of the construct, and it is expressed in Pascals (Pa = N/m^2^) (Bottinelli and Reggiani, 2000; Maganaris et al., 2001; Pirozzi et al., 2013; Miller et al., 2015). CF is proportional to the number of muscle fibers and thus to the cross-sectional area of the construct (Pirozzi et al., 2013). Hence, CF can be easily converted into sF following Equation 1 below.

(1) $$sF=\frac{TwitchorTetanusContractions(N)}{CSA(mm^{2})}$$

**Figure 2.**
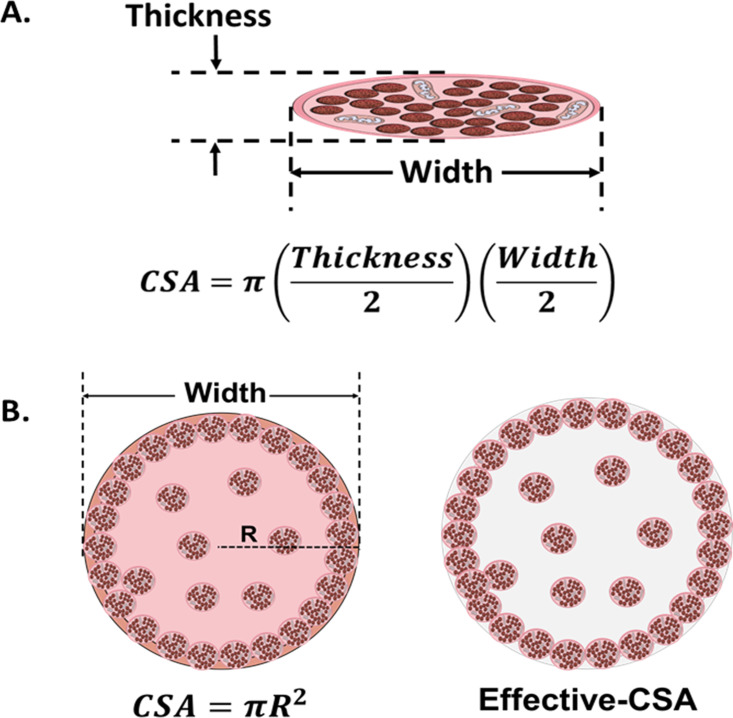
Cross-sectional area (CSA) in 2D and 3D muscle models. (**A**) Myotube CSA estimated as an elliptical shape from the thickness and the width of the cell. (**B**) CSA of 3D muscle constructs can be estimated by approximation to different shapes (circle, in left panel), or calculated from immunohistochemical sections. Effective-CSA is known as the area occupied by myotubes (red area in the right panel).

A major drawback concerning this parameter is the wide diversity of criteria in the literature for defining and quantifying CSA of muscle constructs (Figure 2). In 2D models, CSA can be estimated as a circular shape, or calculated as an elliptical shape from the thickness and the width of the myotubes (Pirozzi et al., 2013; Jeon et al., 2019). As for 3D constructs, the CSA has been approximated to circular (Hinds et al., 2011; Juhas and Bursac, 2014; Nagashima et al., 2020), rectangular (Donnelly et al., 2010), or elliptical (Akiyama et al., 2021) shapes. Moreover, CSA may also be determined from immunohistochemical sections. In this case, most works consider the whole area of the engineered tissue (total CSA). However, non-contractile areas can account for up to 90% of the whole CSA (Sakar et al., 2012; Juhas and Bursac, 2014; Ebrahimi et al., 2021), which has a substantial impact on sF calculations. Thus, other studies consider only the area occupied by the myotubes, which is referred to as effective-CSA (Hinds et al., 2011; Sakar et al., 2012; Sato et al., 2013; Ebrahimi et al., 2021). Other authors normalize CF by cell number (mN/cell) or cell density (mN/cell*mm^–2^), of the muscle construct (Xu et al., 2019).

Studies summarized in Table 1 and Source Data 1 indicate a preference for normalizing CF to the whole CSA (calculated as a circular shape). However, as shown above, the use of sF to compare results across laboratories may turn out to be unreliable due to the large differences in the methodology used to calculate CSA. In addition, tissue processing required for immunohistochemical techniques may entail size artifacts that further affect CSA calculations. Along these lines, a previous study in human isolated fibers claims that diameter measurements present less variability than CSA measurements resulting in a more accurate normalization in N/m instead of sF (Krivickas et al., 2011). We believe that normalizing CF to diameter or to a related parameter (i.e. calculated circular CSA) would allow for a more reliable comparison among different studies, as long as similar contractile areas are verified. In any case, in order to compare sF, it is essential to thoroughly evaluate the method used in each study for CF normalization.

**Table 1.** Maximum contractile force data from in vitro muscle models measured by the three main platforms. Table 1—source data 1.Additional information specific to the experimental setup and stimulation parameters.

	Cell source	Evaluation time	Size	CSA(mm^**2**^)	Twitch contraction		Tetanic contraction		Tetanic-Twitch Ratio*	References
					CF (µN)	sF (kN/m²)	CF (µN)	sF (kN/m²)		
Cantilever deflection	C2C12 myoblasts (mouse)	Day 7	50 μm (Width)^i^ 33 µm (Thickness)*	0.001308*	0.54 ± 0.02	0.41*	1.01 ± 0.14	0.77*	1.87	Fujita et al., 2010
	Rat myoblasts (embryonic)	Day 10–13	22.5 µm (Width)^i^ 10 µm (Thickness)	0.000176*	0.23*	1.3	__	__	__	Wilson et al., 2010
	C2C12 myoblasts (mouse)	Day 6	12.75 µm (Width)* 8–9 µm (Thickness)	0.0000851*	0.80*	9.4 ± 4.6	__	__	__	Sun et al., 2013
	Rat myoblasts (embryonic)	Day 12–14	11.7–23.4 μm (Width) 7.9–13 μm (Thickness)	0.000144*	0.04–0.26 Stoney’s Eq. 0.03–0.18 FEA	0.359–1.70 Stoney’s Eq. 0.168–1.17 FEA	__	__	__	Pirozzi et al., 2013
	Primary human myoblast	Day 23	10 µm (Width)^i^ 6.67 µm (Thickness)*	0.000052*	0.14^g^	2.69*	__	__	__	Smith et al., 2014
	Rat myoblasts (adult)	Day 4–7	16.74 µm (Width)* 11.16 µm (Thickness)^g^	0.000146*	0.17^g^	1.15*	__	__	__	McAleer et al., 2014
	Human myoblasts	Day 3–6	12.11 μm (Width)^g^ 8.07 µm (Thickness)*	0.0000767*	0.78*	9.98^g^	__	__	__	Nesmith et al., 2016 **^#^**
	Human induced pluripotent stem cell	Day 14	11.82 μm (Width)^g^ 10.35 μm (Thickness)^g^	0.0000961*	0.38*	3.98^g^	__	__	__	Badu-Mensah et al., 2020
	Human induced pluripotent stem cell	Day 10–11	9.30 μm (Width)^g^ 6.2 μm (Thickness)*	0.0000452*	0.12 ± 0.02	2.65*	__	__	__	Guo et al., 2020
	Chick myoblasts	3 weeks	11.24 µm (Width)^g^ 7.49 μm (Thickness)*	0.0000661*	1.44*	21.89^g^	3.31*	50^g^	2.28	Santoso et al., 2021
	C2C12 myoblasts (mouse)		16.30 µm (Width)^g^ 10.86 μm (Thickness)*	0.000139*	0.027	0.2^g^	0.018	0.129^g^	0.64	
	Human myoblasts		14.02 µm (Width)^g^ 9.34 μm (Thickness)*	0.0001028*	0.020	0.2^g^	0.019	0.182^g^	0.91	
	Human induced pluripotent stem cell	Day 7–10	22.5 μm (Width)* 15 μm (Thickness)	0.000265*	0.26*	0.986^g^	0.52	1.986^g^	2.01	Al Tanoury et al., 2021
Post deflection	Primary Mouse myoblasts	Day 1–12	2 mm^i^	3.14*	__	__	42.5^g^	13.53*	__	Vandenburgh et al., 2008 **^#^**
	C2C12 myoblasts (mouse)	Day 14	0.14 ± 0.01 mm	0.0125 (active) 0.0012 (effective)	1.4*	0.11 (active)* 1.12 (effective)*	__			Sakar et al., 2012
	C2C12 myoblasts (mouse)	Day 6	0.32 mm^i^	0.079*	__	__	57.5 ± 12.8	0.72*		Shimizu et al., 2017 **^#^**
	Primary human myoblasts	Day 11	0.85 mm*	0.566^i^	79.44^i^	0.14*	428.57^i^	0.76*	5.42	Mills et al., 2019
	Derived-Myoblasts from Human Dermal Fibroblast	Day 4–10	0.30 mm^i^	0.120*	__	__	12.2 ± 5.3	0.10*	__	Shimizu et al., 2020
	Primary human myoblasts	Day 7–14	0.71 mm*	0.395*	__	__	192*	0.49*	__	Afshar et al., 2020
	Immortalized human myoblast	Day 8	0.4 mm	0.125*	__	__	28.5 ± 10.5	0.23	__	Nagashima et al., 2020
	Immortalized human myoblast	Day 7–14	0.47 mm*	0.17 ± 0.03	200 ± 40	1.17	1100 ± 300	6.47	5.52	Hofemeier et al., 2021
	Immortalized human myoblast	Day 10	0.49 mm^i^	0.189*	118.01^g^	0.62*	201.89^g^	1.07*	1.72	Ebrahimi et al., 2021
Force tranducers	Rat myoblasts (adult)	Day 31 ± 4	0.49 ± 0.04 mm	0.188*	215 ± 26	1.14*	440 ± 45	2.9 ± 0.5	2.54	Dennis and Kosnik, Ii, 2000
	Rat myoblast (Extensor digitorum longus)	Day 32 ± 4	0.17 mm*	0.024 ± 0.009	162 ± 125	6.75*	281 ± 218	11.70*	1.73	Dennis et al., 2001
	Rat myoblasts	3 weeks	0.18 ± 0.01 mm	0.0246*	329 ± 26.3	13.37*	805.8 ± 55	32.75	2.45	Huang et al., 2005
	Rat myoblasts	Day 16–18	0.25 mm^g^	0.048*	102^g^	2.12*	212^g^	4.41*	2.08	Larkin et al., 2006
	C2C12 myoblasts (mouse)	Day 5–8	0.21 mm*	0.0978^i^	71.39*	0.73 ± 2.13	86.06*	0.88 ± 0.48	1.20	Fujita et al., 2009 **^#^**
	C2C12 myoblasts (mouse)	Day 2–17	0.2 mm*	0.031*	33.2	1.06	__	__	__	Yamamoto et al., 2011 **^#^**
	Rat myoblasts (neonatal)	Day 14	2.7 ± 0.18 mm (Bundle)	5.72 (Bundle)*	1680 ± 320	0.29* 5.5 ± 0.6 (effective)	2840 ± 500	0.50* 9.4 ± 0.7 (effective)	1.72	Hinds et al., 2011
	C2C12 myoblasts (mouse)	Day 7	0.40 mm	0.13*	18.3 ± 2.4	0.15*	34.5 ± 2.8	0.276*	1.84	Sato et al., 2013 **^#^**
	Rat myoblasts	2 weeks	1.38 mm (Bundle)* 0.9 mm (F-actin+) *	1.50 (Bundle)^g^ 0.63 ± 0.05 (F-actin+)	17830 ± 1000	11.89 (Bundle)* 28.30 (F-actin+)*	28800 ± 930	19.2 (Bundle)* 43.39 ± 3.82 (F-actin+)	1.61	Juhas and Bursac, 2014 **^#^**
	Primary Human myoblast	4 weeks	2.5 mm^i^	4.91*	701^g^	0.14*	1460^g^	0.30*	2.14	Madden et al., 2015
	C2C12 myoblasts (mouse)	Day 14	0.5 ± 0.08 mm	0.19*	81.26^g^	0.42*	151.37^g^	0.79*	1.88	Ikeda et al., 2016 **^#^**
	C2C12 myoblasts (mouse)	3 weeks	0.6 mm^i^	0.28*	166.3 ± 59.4	0.59*	__	__	__	Nakamura et al., 2017
	hPSC derived human myoblasts	2 weeks	0.42 mm^i^	0.14*	140^g^	1.04	402^g^	3.00*	2.88	Rao et al., 2018
	C2C12 myoblasts (mouse)	Day 14	0.98 mm^i^	0.756*	48.39 ± 3.49	0.06	47.74 ± 0.31	0.06	1	Capel et al., 2019
	Primary Human Myoblast	2 weeks	0.62 mm^i^	0.30*	1700 ± 130	5.70*	3400 ± 180	11.40*	2	Khodabukus et al., 2019
	hPSC derived human myoblasts	4 weeks	0.28 mm^i^	0.06*	1393 ± 342	23.21*	2924 ± 517	48.73*	2.09	Xu et al., 2019
	C2C12 myoblasts (mouse)	10 Days	0.99 mm	0.77*	1360 ± 210	1.77*	1930 ± 120	2.50*	1.41	Akiyama et al., 2021
	Primary Human Myoblast	Day 17–19	1.39 mm^i^	1.51*	__	__	175*	0.13*	__	Alave Reyes-Furrer et al., 2021

*Recalculated data; ^**g**^Data extract from a graph; ^**i**^Data extract from an image; ^**#**^Studies with where maximal instantaneous CF data was used.

### CF parameters

CF is affected by its relationship with different parameters, such as the frequency of the contractile stimulus (force-frequency), sarcomere length (force-length), and contraction velocity (force-velocity). Thus, an increase in stimulation frequency causes an increase in CF up to a maximal value (Fitts et al., 1991; Widmaier and Raff, 2008), although prolonged stimulation can lead to tissue fatigue and cause a decrease in CF over time (Grassi et al., 2015). In addition, contraction velocity is inversely related to cross-bridge formations and thus to CF production (Widmaier and Raff, 2008; Lindstedt et al., 2016). Power, defined by the product of CF and contraction velocity, is a measurable outcome of muscle contraction that reflects its efficiency and can be used to assess muscle fatigue (Alcazar et al., 2019). Finally, CF also depends on muscle length. An optimal length (L_0_) is where actin-myosin interactions are maximal and the muscle generates the highest tension (Fitts et al., 1991; Cheng et al., 2014a).

CF assessment should not be limited to determining the peak force value reached during contraction. It is rather preferred to analyze the complete contraction-relaxation profile. In essence, upon stimulation, skeletal muscle may present two types of mechanical profiles, as shown in Figure 1. An isolated contraction, or twitch response, is caused by a one-pulse stimulus that generates a single action potential (Widmaier and Raff, 2008). A tetanic contraction, in turn, is caused by a series of pulses whose frequency determines the contraction profile. Tetanic contraction may be unfused when the stimulation frequency allows the muscle to relax between pulses or fused when the interval between pulses is shorter than the time needed to reach relaxation. In the latter, the muscle exerts maximal CF (Widmaier and Raff, 2008; Wang et al., 2019).

Several parameters can be calculated from twitch and tetanus contraction profiles: The Tetanic-to-Twitch ratio, which compares maximal CF of these profiles, is independent from the size of the construct, and is affected by fatigue and maturation of muscle fibers, among others (Hill et al., 2021; Santoso et al., 2021). Time to peak (TTP) and half-relaxation time (RT_50_) can also be calculated from contraction profiles (Figure 1, lower graphs). These parameters contribute to evaluating contraction kinetics, and have revealed pathological phenotypes, such as impaired contractility in myotubes of ALS patients (Badu-Mensah et al., 2020).

## Techniques for contractile force assessment

Hereafter, the most relevant techniques used to determine and measure CF in engineered skeletal muscle tissues, either for 2D or 3D configurations, are described: (1) cantilever deflection, (2) post deflection, or (3) force transducers. We also discuss alternative or emerging technologies for CF assessment, and provide a detailed comparison of the three main platforms currently in use.

### Cantilever deflection

**Figure 3.**
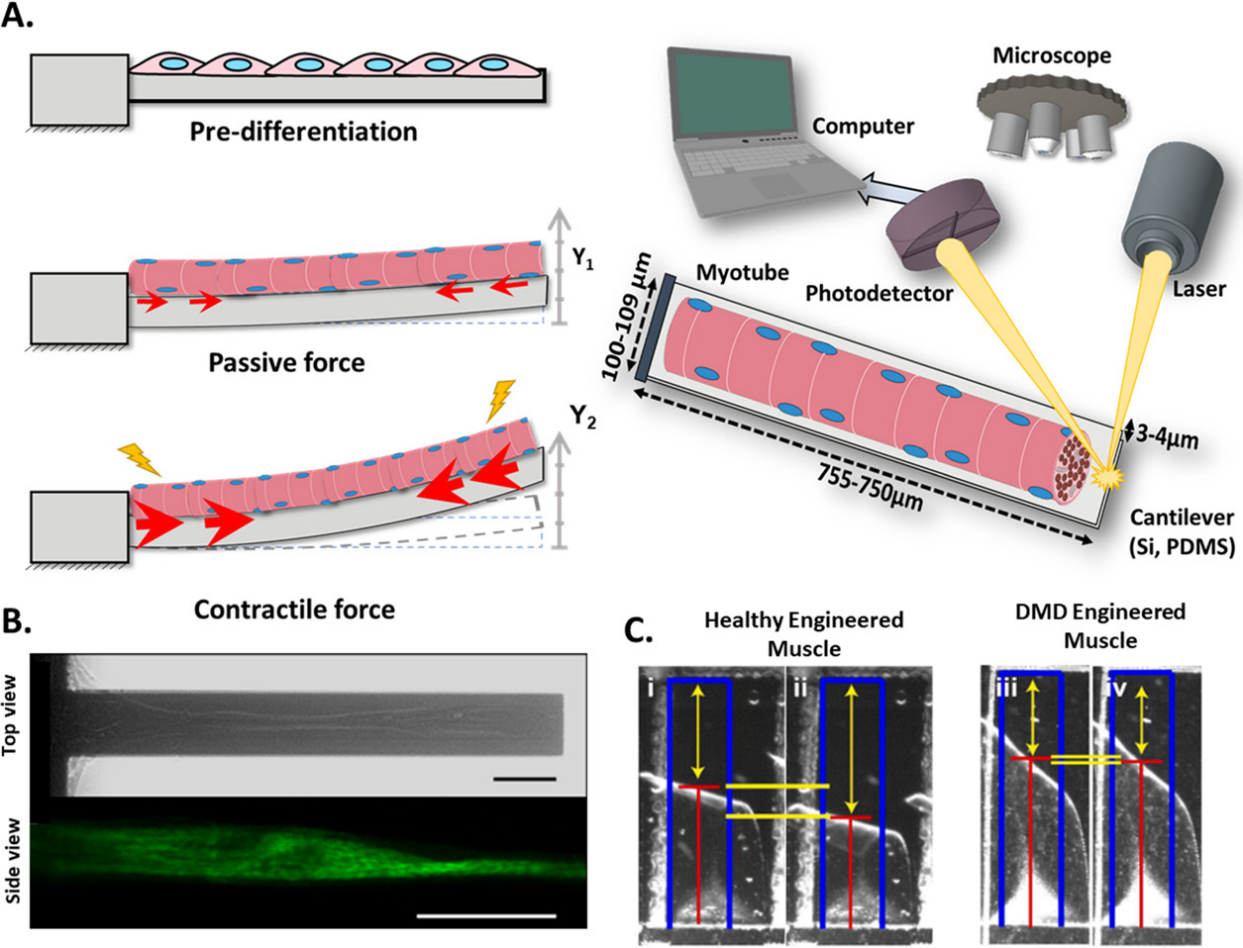
Cantilever deflection setup. (**A**) The beam deflects due to myotube contraction (Left). In this case, cantilever deflection is interrogated by a laser beam and detected using a photodetector (Right). Commonly, cantilever arrays are made of Silicon (Si) or PDMS. Different coatings (FN, laminin, collagen I) have been tested to improve cell attachment and longer culture times. (**B**) Human myotubes on silicon cantilevers in bright field, top view (top) and immunostained for Myosin Heavy chain, side view (above). Scale bar: 50 µm. (**C**) Representative images from healthy and DMD myotubes at baseline (i and iii) and peak stress (ii and iv). Blue rectangles represent film length. Red lines represent the tracking of the film edge. Yellow arrows represent the distance between the projected film length and the unstressed film length. The yellow horizontal lines represent the change in projected film length from baseline stress (top bar) to peak stress (bottom bar).

This technique is based on the deflection of a cantilever beam, which is fixed to one end and free for vertical movement on the other. The cantilever bends proportionally to the CF, and the variation of the height of the free end, or deflection, is measured by a laser and photodetector or image analysis (Wilson et al., 2007; Wilson et al., 2010; Fujita et al., 2010; Sun et al., 2013). Schematics in Figure 3 depict the cantilever setup with its three main states: resting, right after attachment of myoblasts, which are at a pre-differentiation state; initial deflection, due to the passive force exerted by the differentiated myotubes; and increased bending, caused by the CF that myotubes exert under stimuli. This force results in contraction of the cantilever’s upper side and a decrease in its length, while the lower surface of the beam remains relaxed with no change in its length. The differential strain distribution along cantilever thickness causes an upwards bending. Since one end of the cantilever is fixed, the entire bending effect is transformed into an uplift of its free end. Thus, myoblast’s horizontal contraction is transformed into a vertical displacement of the cantilever end.

A backward analysis relating the measured displacement and the compression state of the cantilever allows for CF calculation. This computation can be resolved using Stoney’s equation (Wilson et al., 2007; Smith et al., 2014; Santoso et al., 2021), Finite Elements Analysis (FEA) (Pirozzi et al., 2013), or analytical solutions for the curvature radius (Wilson et al., 2007; Nesmith et al., 2016; Al Tanoury et al., 2021). The first two methods are best suited for a silicon cantilever setup, and according to Pirozzi and colleagues, Stoney’s equation provides enough accuracy for most applications. The FEA analysis is more mechanically rigorous but also computationally more complex. In this case, a laser-photodetector system is used to measure the vertical displacements, which are relatively small. The third approach is most appropriate for a polymeric film-like substrate in which image analysis will provide a change in the radius of curvature of the film. These materials allow larger deformations of the substrates (almost rolling) than the stiffer cantilevers. In either case, a thorough mechanical characterization of the system (e.g. thickness, flexibility) is required in order to feed the previous models.

These calculations assume that myotubes and substrates behave as one continuous solid element rather than two independent ones. Thus, myotubes need to be perfectly attached to the substrate. Special care needs to be taken to identify potential inhomogeneities within the culture or misalignments from the deflection axis since any slight deviation could render significant differences in CF assessment (Wilson et al., 2010). Techniques such as microcontact printing have been proposed to address these challenges, providing alignment and direct cell adhesion to the cantilever by using Collagen I (Guo et al., 2020), DETA (Wilson et al., 2010), or fibronectin (Nesmith et al., 2016; Sun et al., 2013). The use of coatings like DETA (Wilson et al., 2010) promotes long-term cell attachment (up to 21 days), which allows higher maturation of muscle constructs. Furthermore, the use of laminin and cell-tak, a tissue adhesive, enables evaluation of tetanic contractions in C2C12 myotubes with a reported tetanic-to-twitch ratio >1.5 (Fujita et al., 2010).

As a representative example, Figure 3B shows a top view (bright field) and a side view (fluorescence) image of a silicon cantilever with one single human myotube atop, and Figure 3C shows a representative image of a polymeric film-like structure with healthy and dystrophic myotubes under stimulation.

One important advantage of this technique is that there is no manipulation of the tissue at any time; hence, we consider this technique to be non-invasive and with high throughput potential. This 2D platform has been used to test different cell sources such as C2C12 (Fujita et al., 2010; Shimizu et al., 2010; Yamamoto et al., 2011; Pirozzi et al., 2013; Sun et al., 2013), myocytes from rats (McAleer et al., 2014; Wilson et al., 2010) or chicken (Santoso et al., 2021), human-induced pluripotent stem cells (hiPSCs) (Badu-Mensah et al., 2020; Guo et al., 2020), or primary human myoblast (Smith et al., 2014; Nesmith et al., 2016). Moreover, muscle disease models such as DMD (Nesmith et al., 2016; Al Tanoury et al., 2021) and amyotrophic lateral sclerosis (ALS) Badu-Mensah et al., 2020 have been evaluated on this platform, providing relevant information about the pathological mechanisms, and even testing novel treatments.

### Post deflection

This technique is based on the quantification of the deflection of a pair of vertical microposts (µposts), due to contraction of the myobundle (Vandenburgh et al., 2008). The tissue construct, which is attached to the top part of the µposts, pulls them together during contraction, causing a horizontal displacement. This effect enables the calculation of the CF from equation (2), where R is the µpost pillar radius, L the length (height), E the elastic modulus, δ the µpost top displacement, and F is the CF of the muscle (Vandenburgh et al., 2008; Shimizu et al., 2017; Nagashima et al., 2020).

(2) $$F=\frac{3\pi ER^{4}\delta }{4L^{3}}$$

This equation corresponds to the deflection of a beam with a circular section. According to the classical principles of material science, the application of this expression assumes slender µpost profiles, exertion of myobundle force at the top of µposts (instead of a larger region as depicted in Figure 3), and a linear cantilever deflection in response to the exerted force. This is true if the stiffness of the µpost is at certain ranges, according to Sakar et al., 2012. They reported that an overly flexible cantilever might result in non-linear responses. Also, the effect of the top cap on the bending behavior of the µpost is negligible. However, if one would require higher fidelity on these calculations, finite element analysis could be used to include the thickness of the myobundle at the attachment site as well as any other structural feature. For example, the spring constant of the µposts was calculated with this methodology by including its exact geometry and the material properties (Hofemeier et al., 2021). In any case, a thorough characterization of the µposts is required prior to establishing the cell culture (i.e. µposts stiffness and shape [Vandenburgh et al., 2008; Christensen et al., 2020]).

**Figure 4.**
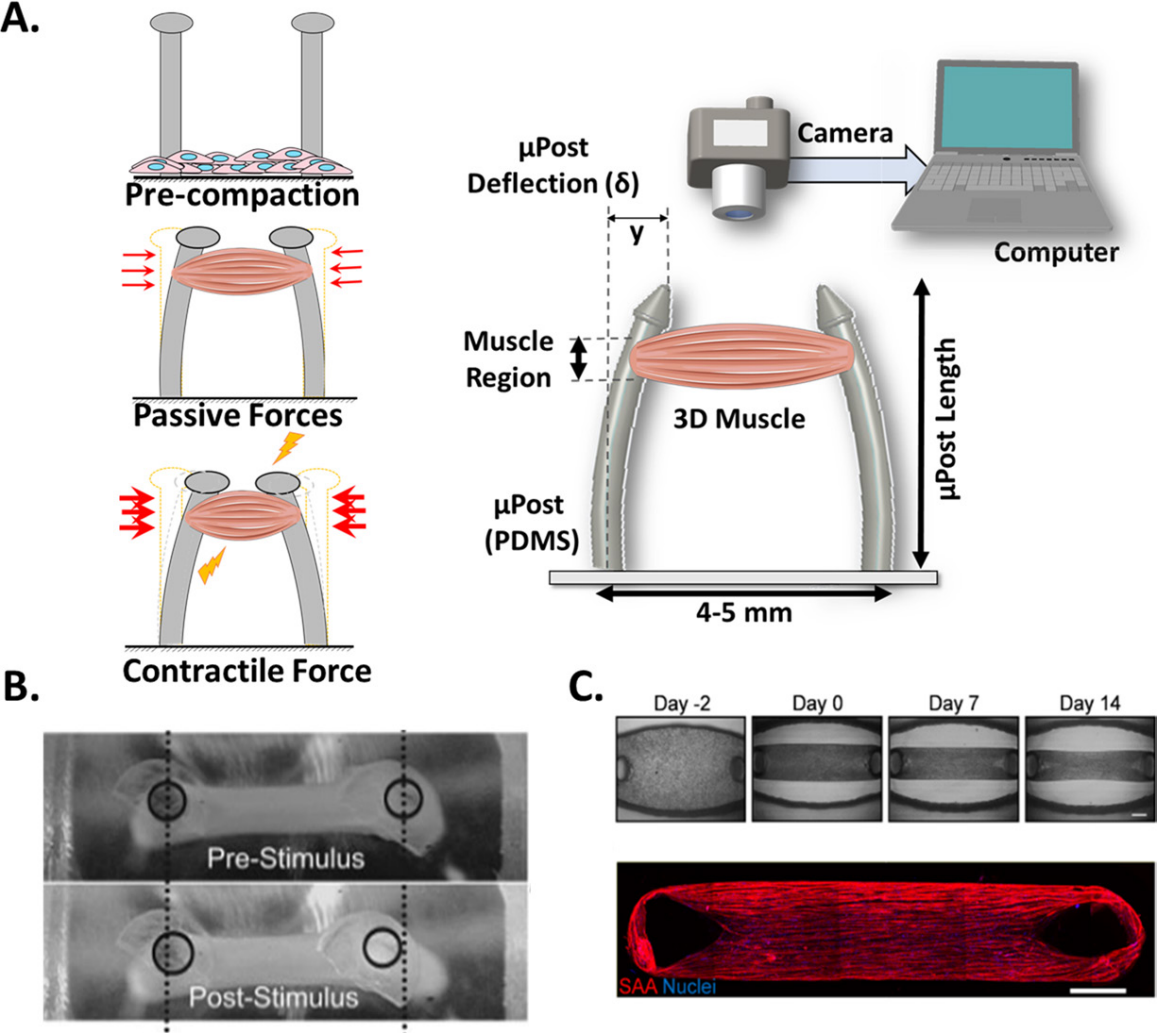
Post Deflection features. (**A**) In vitro skeletal muscle is grown between two micropost which serve as anchors (tendons). As muscle contracts in response to a stimulus, posts bend proportionally. By tracking these displacements and knowing the mechanical characteristics of the platform, the force exerted by the muscle can be quantified. (**B**) Micropost displacement due to miniature bioartificial muscle (mBAM) contraction in response to a maximum tetanic electrical stimulus. Scale bar: 100 µm. (**C**) Formation of human skeletal muscle micro-tissue (hMMTs). Phase-contrast images depicting the remodeling of the ECM by human myoblast over time. Muscle construct immunostained (2 weeks) for sarcomeric α-actinin (SAA, red) and counterstained with DRAQ5 (1, 5-bis{[2-(di-methylamino)ethyl]amino}–4, 8-dihydroxyanthracene-9, 10-dione) nuclear stain in blue. Scale bar: 500 µm. Reprinted from Figure 2A and C from Afshar et al., 2020.

Commonly, 3D skeletal muscle constructs are generated by embedding myoblasts in hydrogels (i.e. collagen, matrigel, fibrinogen) that simulate the native extracellular matrix (ECM) structure (Pollot et al., 2018) and provide the appropriate environment to develop the muscle structure. A casting process of the hydrogel and myoblasts allow the formation of the myobundle around the integrated µposts. Figure 4A (right) shows a graphical representation of this system, where the post deflection is assessed by a video camera. Figure 4A (Left) provide detailed representation of the three main states of the µposts: (1) resting, before the gelation or polymerization of the hydrogel, (2) initial deflection, due to the passive force exerted by the newly formed construct, and (3) increased deflection, caused by a contraction of the construct. The uniaxial passive force within the hydrogel (started at step 2) will promote myoblast alignment (Vandenburgh et al., 1996; Ruedinger et al., 2015; Thorrez et al., 2018) and myotube formation. Figure 4B shows a representative image of this displacement recorded from a top view before and after stimulation, and Figure 4C shows a typical sequence of the myobundle formation and an immunostaining image below, to show the alignment of the fibers and the formation of myotubes.

Vandenburgh and colleagues pioneered the development of this type of experimental setup in 2008 (Vandenburgh et al., 2008). Since then, many studies have been published using this platform, ranging from analysis of µpost design (Christensen et al., 2020), cellular sources (Yamamoto et al., 2009; Shimizu et al., 2020; Nagashima et al., 2020; Yoshioka et al., 2021a), drug screening assays (Afshar et al., 2020; Nagashima et al., 2020; Yoshioka et al., 2020) and passive forces (Agrawal et al., 2017), to the evaluation of contractility recovery following atrophy (Shimizu et al., 2017) or in a ALS-motor unit (Osaki et al., 2018b, Osaki et al., 2018a).

One advantage of this setup is that CF can be evaluated throughout the development of the muscle construct without causing disturbance, since information may be collected from the same sample as many times as required. Interestingly and perhaps due to its simplicity, this technique has encouraged the development of commercial platforms currently used to develop bioengineering cardiac tissues (Hansen et al., 2010). The major challenge is to track the deflection of µPosts accurately. In this line, fluorescent microbeads have been proposed as a means to easily follow µPost displacements (Sakar et al., 2012). For the above-mentioned reasons, we consider the post deflection method to be non-invasive, with high-throughput potential.

Many authors have adopted this experimental setup in combination with different stimulation protocols in order to enhance tissue maturation and induce contraction. For example, by chronical optical stimulation with ChR2, an increase of twitch and tetanic CF was observed in C2C12 (Sakar et al., 2012) and in human skeletal micro muscles (hµMs) (Mills et al., 2019). Alternatively, Kim and colleagues have used co-stimulation (mechanical and electrical) in a fascicle-like muscle model (eSMT), aiming ECM remodeling and thus CF improvement (Kim et al., 2019).

### Force transducers

**Figure 5.**
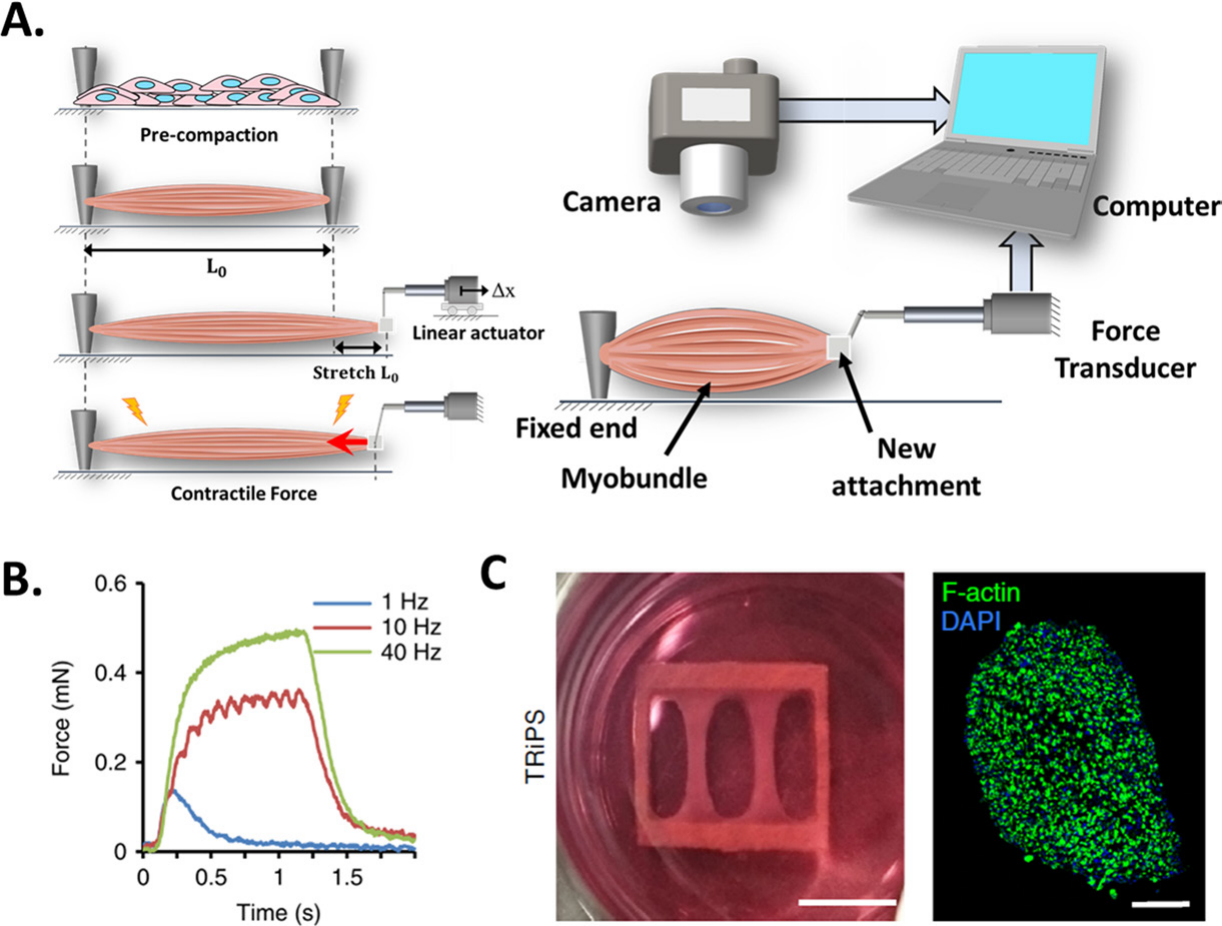
Force transducers. (**A**) In vitro 3D tissue is grown between two anchors. To assess contraction force, one of its sides is connected to a force transducer which will evaluate the force exerted by the muscle due to stimuli. (**B**) Representative contractile properties of hPSC-derived iSKM bundles. TRiPS-derived bundle (4 weeks) shows increases in contractile force with an increase of stimulation frequency up to the formation of tetanic contraction. Specific force and tetanic-to-twitch ratio of H9 and TRiPS-derived bundle (2 weeks) and (**C**) (Left) two-week differentiated iSKM bundles pair anchored within a nylon frame. (Right) Representative immunostaining of dense, uniformly distributed myotubes in bundle-CSA. Panel B reprinted from Figures 3A, B and 4A from Rao et al., 2018.

This technique is based on the use of a high-resolution force transducer coupled to the end of an engineered muscle (the other end is fixed to the substrate, as illustrated in Figure 5A). As the tissue contracts, it pulls from the transducer, allowing CF to be measured. The transducer converts the CF into a digital signal that can be recorded by a computer with the appropriate instrumentation.

Unlike the previous techniques, this setup only allows to perform endpoint measurements and requires manipulation of the 3D construct (invasive and low throughput), which may affect tissue structure and CF performance. Figure 5A (left) represents a step-by-step process of this setup. In short, the engineered muscle is grown in a specific bioreactor and then transferred to another setup where it is coupled to the force transducer (Alave Reyes-Furrer et al., 2021). This procedure is quite delicate as the muscle is prone to breakage. A linear actuator is then used to locate and set the construct to its original length and stretch the tissue above its baseline length (L_0_). Before taking any measurement, the construct may require time to stabilize and reacquire its resting state. Otherwise, tissue performance would be affected by the stress generated by the exchange of platforms.

Figure 5B shows a representative graph where the muscular construct has been subjected to a series of stimuli in order to determine its response to pulse frequency from twitch (1 Hz) to fused tetanic response (40 Hz). And Figure 5C presents a descriptive photograph of 2-week differentiated induced skeletal muscle bundles (iSKM) anchored within a nylon frame and a typical representative immunostaining of dense, uniformly distributed myotubes in bundle-CSA.

Researchers have used this platform to evaluate several conditions on 3D muscle constructs, such as cell sources (Dennis et al., 2001; Madden et al., 2015; Maffioletti et al., 2018; Rao et al., 2018), co-cultures (Larkin et al., 2006; Juhas et al., 2018) and media supplements (Fujita et al., 2009; Xu et al., 2019), as well as to perform drug screening assays (Ikeda et al., 2017; Zhang et al., 2018; Khodabukus et al., 2020). For example, Dennis and Kosnik generated three-dimensional skeletal muscle constructs from adult rats (myooids), to assess different CF parameters such as rheobase and chronaxie (Dennis and Kosnik, Ii, 2000), while Larkin et al., and Dhawan et al. evaluated the effect of muscle innervation on CF. Here, as expected the nerve-muscle constructs generated greated tetanic CF in vitro (212 µm vs 90 µN) and in vivo (649 ± 228µN vs 124 ± 31µN), respectively (Larkin et al., 2006; Dhawan et al., 2007).

### Alternative and emerging technologies

Although force transducers, cantilevers, and post deflection are the most commonly used technologies to assess the production of CF in vitro, in this section we discuss several techniques that are being explored as an alternative way to quantify contraction kinetics: Traction force microscopy, contractility analysis, FRET, and microelectrode arrays (MEA).

Traction Force Microscopy (TFM) enables the measurement of local forces together with their directionality through the displacement tracking of micropillars or fluorescent microbeads embedded in elastic substrates (Ribeiro et al., 2016; Lemke and Schnorrer, 2017). This technique also allows the determination of traction forces along the entire length of the myotube, and throughout its differentiation process (Li et al., 2008). It is, thus, a non-invasive approach, which is also compatible with high-resolution microscopy. However, it presents some limitations inherent to the setup, such as the requirement of relatively low cell densities to track forces correctly, which may impact the size of de novo generated myotubes (Bruegmann et al., 2010). Thus, some authors use TFM with isolated native muscle fibers to overcome this limitation (Rausch et al., 2020). Inspired by this method other authors have determined the tensional status of hydrogels-containing human or C2C12 myotubes, by tracking deformable polyacrylamide fluorescent microbeads throughout the culture compaction and development (Hofemeier et al., 2021), or by detecting deformation of the substrate through Particle Imaging Velocity (Zhang et al., 2018; Cheesbrough et al., 2022).

Contractility-Pixel analysis relies on detecting changes in the position of the tissue by measuring the change of pixel intensity over time for a given region (Fujita et al., 2007; Kaji et al., 2010; Langhammer et al., 2010; Furuhashi et al., 2021). Overall, this technique does not provide a direct measure of CF, but instead, it resolves contraction and relaxation velocities and enables generation of curves, where contractility is usually presented as % of movement or as arbitrary units (Sala et al., 2018). Similar to TFM, this technique allows monitoring throughout culture development and it is also non-invasive. In contrast, image analysis is compatible with high-density cultures and even co-cultures. This method has been applied for drug-screening assays, and for the evaluation of contractility of individual cells and myotubes, to monolayers and 3D constructs (Kaji et al., 2010; Shimizu et al., 2015; Osaki et al., 2018a; Takahashi et al., 2018; Afshar Bakooshli et al., 2019; Vila et al., 2021).

Forster Resonance Energy Transfer (FRET) enables the measurement of contractile forces at the molecular level (pico Newtons, pN) based on changes of fluorescent emission upon the interaction of a pair of fluorescent probes (Grashoff et al., 2010; Shrestha et al., 2015; Wu et al., 2020). Changes in distance, stretching ( < 10–12 nm) tension, or orientation between probes modulates energy transfer, and thus the contraction measurements (Cost et al., 2015; Algar et al., 2019; Kaur and Dhakal, 2020). Interestingly, this method allows intracellular CF monitoring during culture development as it is non-invasive. However, it presents some specific challenges, such as an appropriate calibration to relate the light intensity with contraction force (Gates et al., 2019), the spatial-temporal resolution needed during contraction, and the lifespan of the fluorescence probes regarding the time required for differentiation processes. This procedure has been already applied to the evaluation of internal forces on F-actin and actinin using HEK-293 cells (Guo et al., 2014), in evaluating structural changes in the force-generating lever arm in myosin V (Gunther et al., 2020). It has also been applied in vivo to study the relationship between calcium handling and contraction in zebrafish beating hearts using the ratiometric biosensor Twitch-4 (Salgado-Almario et al., 2022). While further studies are needed to implement FRET for CF assessment of engineered skeletal muscles, we expect that it will become a valuable technique, complementary to the main techniques currently applied (Komatsubara et al., 2015).

Finally, Microelectrodes arrays (MEAs) systems have been used to characterize the electrical activity of neuron, cardiac or skeletal muscle cellular networks with a high spatial resolution. (Massobrio et al., 2015; Rabieh et al., 2016; Pasquarelli, 2021). These microelectrode arrays are able to record the bioelectric signals generated by the culture and/or stimulate them. Indeed, electrical stimulation has been already proven to be effective to promote myotube formation (Langhammer et al., 2013). But more importantly, this MEAs system can provide impedance monitoring to assess contractility, through the analysis of cell-microelectrode interactions. With each contraction, the changes of the shape of myotubes and the microelectrodes coverage, produces a perturbation in the AC current between pairs of electrodes proportional to the myotube movement allowing contraction analysis (Axion Biosystems, 2021). Even though this method does not provide quantitative CF measurements, it would provide relevant functional information of contraction kinetics.

### CF platform comparison

**Figure 6.**
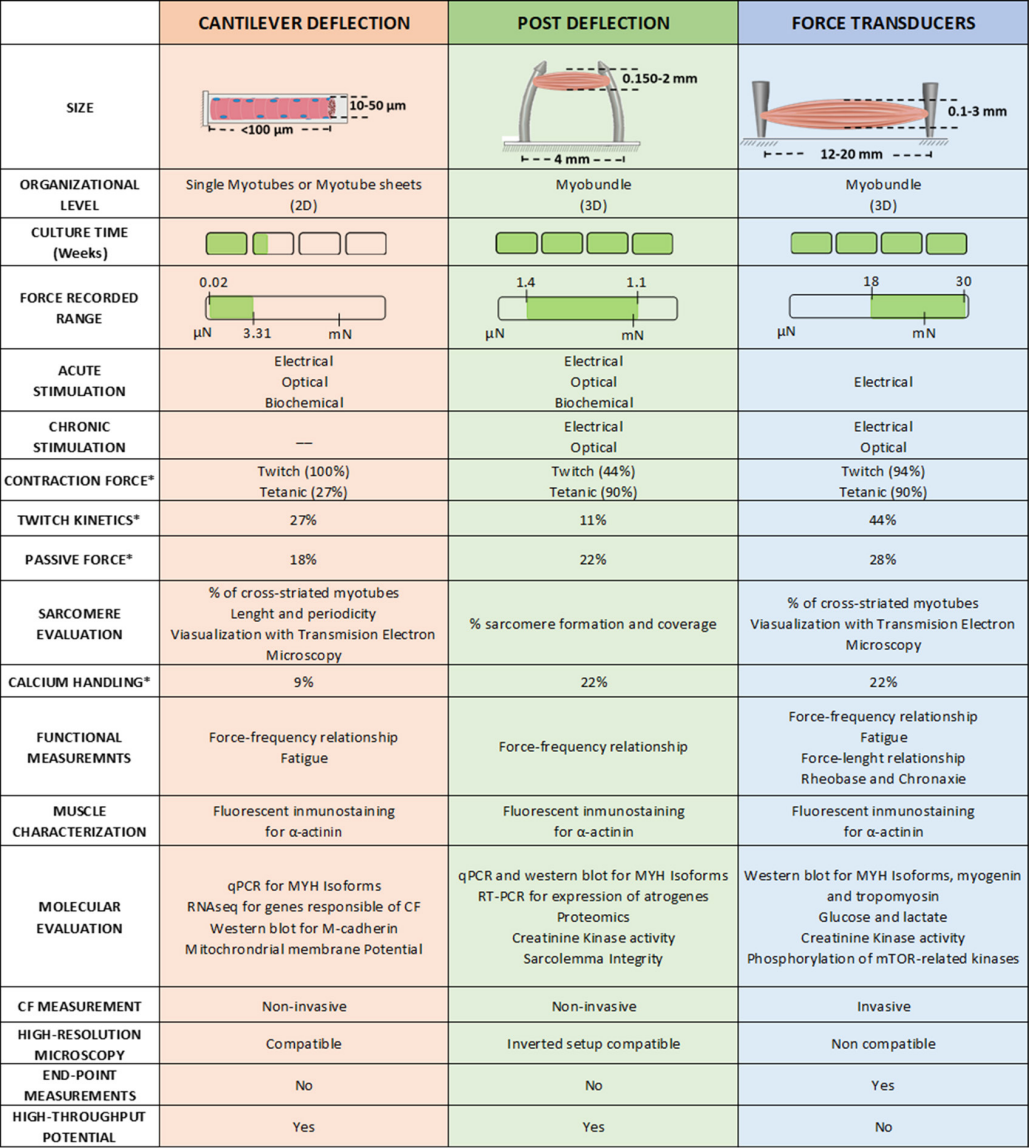
Overview of the different techniques used to measure contractile force in vitro. * Represents de % of studies that have performed this measurement. Figure 6—source data 1.Editable version of Figure 6.

There are a number of significant differences among the three main CF assessment methodologies, which are summarized in Figure 6. Generally, cantilevers are best suited for 2D models, myotubes, or muscular films, while µposts and force transducers are designed only for 3D tissue constructs, also known as myobundles. Besides the size, 2D cultures are usually kept in culture for a shorter time and present a risk of delamination, which increases with contractile activity. This partially explains the lower percentage of studies that have performed tetanic stimulation in 2D muscle models. In contrast, 3D models are cultured over longer periods, and present a lower risk of detachment from the µposts or anchoring structures. These 3D constructs are more resilient to chronic stimulation and better withstand tetanic stimulation (up to 90% of studies present this analysis). All three setups have been combined with electrical stimulation, typically by introducing platinum, gold, or carbon electrodes. However, optical and biochemical stimulation was only reported in cantilever and post deflection.

The measured force range in Newtons for each platform is also significantly different, being 0.02–3.31 µN for the cantilever, 1.4 µN – 1.1 mN for µposts, and 18 µN – 30 mN for the transducer. These values are consistent with the sizes of the constructs each platform can accommodate. Figure 6 also presents the ratio of studies that presented the values of passive force (the contractile force at a resting state without stimulation) and twitch kinetics (PA, TTP, RT_50_). In addition, there are a number of contractile functional measurements that have been implemented in each platform: that is calcium handling, force-frequency relationship, fatigue, or force-length relationship. However, insufficient data is available to perform a fair comparison between studies (less than 20% of the studies evaluated these characteristics).

Regarding other functional outcomes, Figure 6 includes information about sarcomere evaluation, immunofluorescent imaging of α-actinin, and molecular expression (including genomic and proteomic). Most of these analyses are covered in all three setups, but the availability of biological material greatly differs depending on the setup. 3D muscle constructs provide larger amounts of tissue for analysis, enabling more extensive evaluations and assessments of scarcely expressed molecules.

Other characteristics unique to each setup need to be considered: Cantilever and posts deflection are potentially considered high throughput techniques, while force transducer platforms are not yet ready to handle many samples in parallel. Similarly, the first two are also non-invasive since there is no manipulation of the tissues to obtain the CF and allow for continuous monitoring of the evolution of the culture. The force transducer setup requires getting the myobundle or muscle construct attached to the sensor, and therefore, it becomes an end-point measurement. However, handling the myobundle allows control of the initial length of the myobundle prior to stimulation, enabling the analysis of additional variables. This feature is of most relevance as it can directly impact the force generated by the construct can generate. Another important feature is the compatibility of these techniques with high-resolution microscopy. While a 2D setup is easily integrated, 3D constructs have major limitations and require modification of the culturing setup. One should finally consider the complexity of the culture setup and the measuring platform, its cost, the access to appropriate facilities, as well as the required training/experience of the personnel operating these measurements. For instance, cantilever or µposts fabrication is quite complex since it requires microfabrication facilities or specific fabrication equipment, while no specific culturing protocols are required. On the contrary, the force transducer platform requires straightforward instrumentation, but it requires expert skills to manipulate the tissue construct correctly.

## Contractile force data analysis

The previous section gathers a summary of the most relevant techniques used to quantify CF in vitro. There is a distinctive approach for 2D and 3D tissue models and the different methodologies used to assess CF in each of them. This section provides a uniform analysis of published data using the three methods described, enabling comparison of contractility across different models. It also provides insights into the development of disease models and evaluation of the effect of several pharmacological treatments.

### Analysis criteria

It is relatively common to find CF values with different metrics or without proper clarification of morphological parameters such as sample width/thickness or CSA. This indetermination hinders a straightforward comparison between data collected using the different methodologies. Table 1 compares the morphological information (diameter and CSA) and the CF and sF of different healthy muscle constructs. CF data was directly extracted from the studies, time-average measurements were selected for both twitch and tetanus contraction unless these data were not provided in the study (noted with **^#^**). Maximum or instantaneous CF values were used as a necessary replacement in those cases. Additionally, it was often necessary to recalculate other parameters from the information provided by the authors (graphs or images), since it was either missing or provided in different units. Values that were recalculated are noted with an asterisk (*) in the table. To calculate the CF values, we have assumed the following premises:

1. Determination of the CSA (mm^2^): For 2D models we used the width (diameter) and thickness of the tissue, assuming an elliptical shape ($CSA=\pi {\left(width/2\right)}^{}{\left(thickness/2\right)}^{}$). Calculations were based on diameter measurement assuming that myotube thickness is 1.5 times lower than diameter. For the 3D models, we worked with the circular cross-section of the sample (CSA = $\pi R^{2}$ , where R is the radius of the construct). Note that for the comparison among studies, all data have been normalized to the whole CSA (Figure 2).
2. If the authors present the sF directly rather than CF from twitch or tetanus, a backward calculation was made to ensure the comparison for either the CSA or CF.
3. Tetanic-to-Twitch Ratio was calculated from sF data within the same study (sF_Te_ / sF_Tw_), except for the post deflection experiments with C2C12 myotubes, where data unavailability forced the use of sF_Te_ and sF_Tw_ data from different studies.

#### Muscle construct size and composition

The size of myotubes in vitro is smaller than the size of muscle cells in the native muscle tissue, commonly known as muscle fibers (Figure 1). While in the muscle fibers size ranges from 10 to 100 µm (King et al., 2004; Powell et al., 2002), the size of the myotubes in the cantilever deflection was 33.15 ± 23.831 µm and 13.29 ± 4.810 µm for C2C12 and human, respectively. This in vitro data is clearly within the lower range of the scale and could be related to the lack of maturation of the tissue.

**Figure 7.**
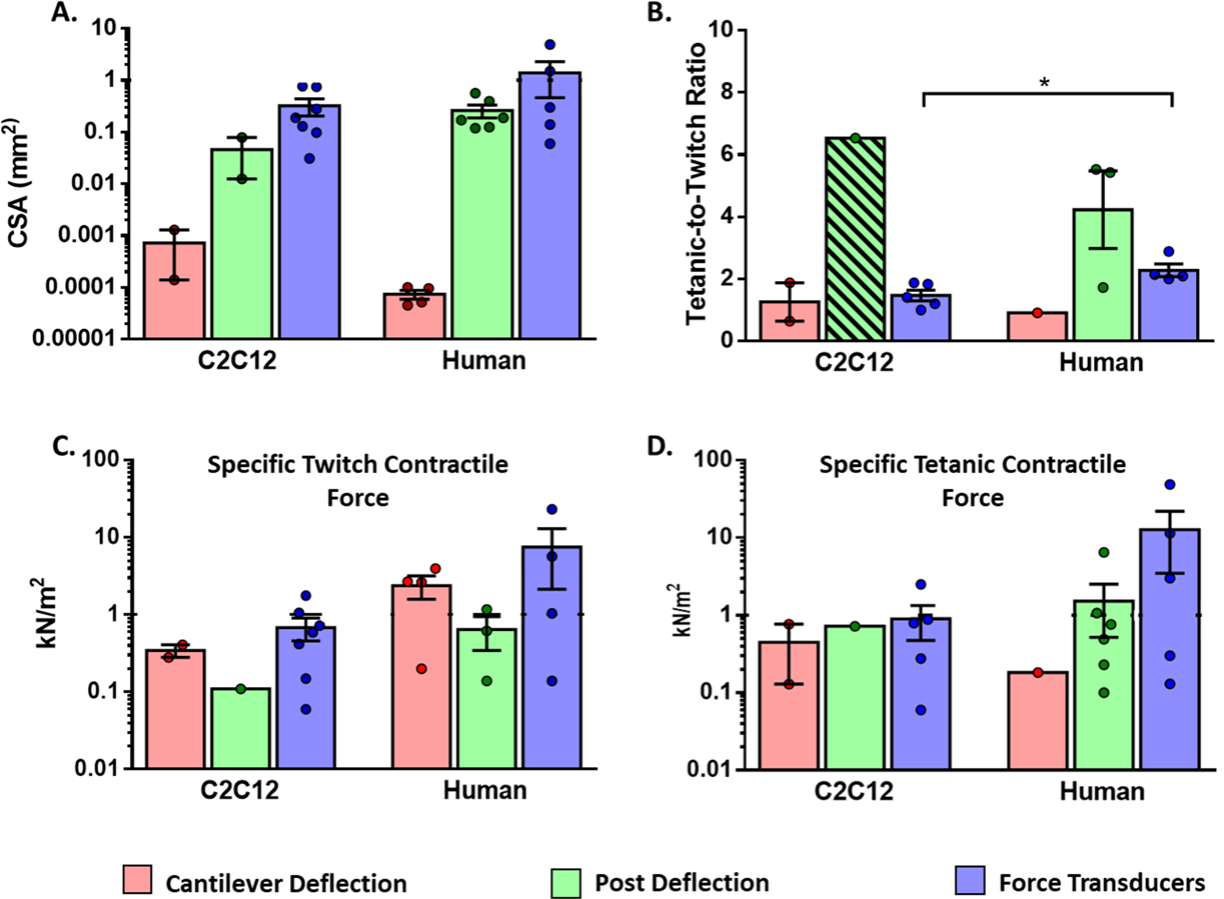
Functional characteristics of in vitro 2D and 3D skeletal muscle tissues from C2C12 and human sources (immortalized, iPSC and primary myoblast). (**A**) Whole cross-sectional area (CSA) of muscle tissues. (**B**) Tetanic-to-Twitch ratio was calculated from data within the same study, except for bar with a diagonal pattern in post deflection, which was calculated from two different studies. (**C**) Twitch and Tetanic specific force measure in the three platforms for C2C12 constructs and (**D**) Human source. Data is presented as mean ± SEM. *p < 0.05, unpaired t-Test.

In 3D muscle constructs in vitro, the size is limited by gas diffusion and, thus, it is difficult to scale up. Diameters of the engineered tissues range between 0.14–2 mm for post deflection and 0.17–3 mm for force transducers. While as shown in Figure 7A, the average-CSA ranges between 0.001–1 mm^2^ for post deflection, and 0.0001–5 mm^2^ for force transducers. Of note, the length of muscle constructs, which is independent of the diameter, is substantially higher in the force transducer platform (12–20 mm) compared to the one in µposts (around 4 mm) (Vandenburgh et al., 2008; Gholobova et al., 2020b).

The dimensions of these constructs are affected by several variables such as the size of the culture platform (i.e. mold dimensions), hydrogel composition, and compaction (collagen vs. fibrinogen and matrigel) (Hinds et al., 2011; Pollot et al., 2018), culture protocols (i.e. stimulation during myogenesis), and cell culture density (Cvetkovic et al., 2014; Yoshioka et al., 2021a).

The scaffold composition, the type of proteins and their concentration, are also essential for tissue development and contraction performance. For example, time-to-tetanic peak was found almost two-times higher in 4 mg/ml fibrin-based hydrogels than in 2 mg/ml hydrogels. Additionally, when comparing fibrin with collagen I hydrogels, the latter exhibited lower twitch CF, tetanic CF, and tetanic-to-twitch ratio compared to fibrin hydrogels (Hinds et al., 2011). This can be partially explained by the elastic modulus of the hydrogel and the pore size. Collagen presents higher stiffness than fibrin, thus affecting the mechanical response of the tissue. The muscular cells need to overcome the elastic resistance provided by the hydrogel to exert proper contraction. On top of that collagen hydrogels present the smallest pore size and a higher degree of crosslinking fibers. Although this characteristic promotes differentiation, it also hinders cell migration (Pollot et al., 2018).

However, the core common issue in these 3D models is the number of myotubes and their localization within the scaffold. The cross-sectional area of native skeletal muscle presents a high and homogeneously distributed density of muscle fibers per area area (King et al., 2004). In contrast, histological studies of in vitro models have shown that myotubes are mainly in the outer ring of the constructs rather than homogeneously distributed along the cross-section (Nakamura et al., 2017). Thus, up to 90% of the CSA could be constituted by non-contractile tissue (Juhas and Bursac, 2014; Sakar et al., 2012; Akiyama et al., 2021; Ebrahimi et al., 2021), likely due to the lack of proper diffusion of nutrients and oxygen within the in vitro tissue (van der Schaft et al., 2011).

Several approaches such as tissue vascularization are being explored to overcome this hindrance (Gholobova et al., 2020a). For example, the use of porous biodegradable polymers promotes muscle differentiation and development of pre-vascular vessel-like structures (Levenberg et al., 2005). Other approaches include the use of sacrificial templates with thermo-responsive materials mimicking the vessel system (Wan et al., 2020), the development of aligned prevascularized muscles (van der Schaft et al., 2011), and the stimulation of angiogenesis and myogenesis through the exposure of myobundles to close-by engineered capillary networks (Osaki et al., 2018a).

### Specific contractile force analysis

The performance of tissue constructs was analyzed by using Twitch Specific Force (T_w_-sF), Tetanic Specific Force (T_e_-sF), and tetanic-to-twitch ratio parameters, which can be extracted or calculated from most studies (Table 1, Figure 7). In order to perform a consistent comparison, we have used the whole CSA (Figure 7A) to calculate sF, and segregated the data into two groups: muscle tissues generated with human myotubes or C2C12 myotubes. Of note, in this analysis human myotubes were generated from immortalized and primary human myoblasts, as well as human induced pluripotent stem cells (hPSC).

CSA data of muscle constructs (Figure 7A) was used to calculate sF generated in the different platforms (Figure 7C and D). These graphs show a vast data dispersion that required logarithmic scales. High dispersion and low data numbers prevented reaching statistical power for proper comparison among the different platforms. In any case, sF values of muscle constructs were much smaller (0.06–50 kN/m^2^) compared to the ones reported for native muscle, with maximal sF in the range of 100–700 kN/m^2^ (Maganaris et al., 2001; Urbanchek et al., 2001; Miller et al., 2015; Jeon et al., 2019).

On the contrary, tetanic-to-twitch data in Figure 7B presented low variability. This parameter was calculated from T_e_-sF and T_w_-sF data within the same study, except for the case of C2C12 in µposts that was calculated from two different studies, due to lack of data availability. Interestingly, for the force transducer platform, we found that the tetanic-to-twitch ratio was significantly higher in human muscle compared to C2C12 muscle constructs (unpaired t-test, p < 0.05). We are uncertain about the relevance of this finding, but it is likely an intrinsic feature related to the species since gender-specific differences have been found for this parameter in native mouse muscles (Lafoux et al., 2020). Of note, this parameter is independent of CSA and provides a functional measurement that could be used to compare different studies regardless of the construct size or platform used.

### Contractile force evaluation in disease models

Evaluation of CF in these platforms has also been successfully used to characterize the phenotype of muscle disease models. Table 2 shows a collection of the most relevant studies where CF is assessed for different drug-induced models or patient-dystrophic models. Remarkably, many of the studies have used human cell lines or primary cells from donor patients. The CF analysis confirmed that the disease engineered tissues exhibit an overall lower sF and poorer morphological characteristics compared to their healthy controls.

**Table 2.** Summary of several skeletal muscle disease models in post deflection and force transducer platforms. Key parameters like drugs, change in CF (%∆CF) and other observed effects are detailed.

Disease model	Drug	Platform	Cell source	%∆CF(Dose)	Observed effect	Reference
Atrophy	Dexamethasone	Post deflection	C2C12 myoblast (mouse)	–53% (100 µM)	Increase in the expression of Atrogin-1 (2.6) and MuRF-1 (2.2) Decrease in number of fibers with striation patterns (20% vs 8%)	Shimizu et al., 2017
			Immortalized human myogenic cells	–57% (100 µM)	Increase in the expression of Atrogin-1 and MuRF-1. Expression of FOXO3 and KLF15	Nagashima et al., 2020
			Primary Human myoblast	–85% (10 nM)	Dose-dependent decrease in myotube width	Afshar et al., 2020
			C2C12 myoblast (mouse)	–48% (1 mM)		Yoshioka et al., 2020
		Force transducer	Primary Human myoblast	–67% (25 µM)	Decrease in myotube diameter (25 µM, 12%) Decrease in effective-CSA (60%) Decrease in injury biomarkers CK and LDH	Khodabukus et al., 2020
			C2C12 myoblast (mouse)	–70% (100 µM)	Decrease in myotube-CSA (37%)	Aguilar-Agon et al., 2021
Hypertrophy	IGF-1	Force transducer	Rat myoblast	+ 31% (75 ng)	Increase in CF (75 ng, 31%) Slow Time to peak twitch force (25 ng, 26%)	Huang et al., 2005
			Primary Human myoblast	+ 28% (0.5 mg/ml)	Increase in myotube diameter (0.5 mg/ml, 21%) Increase in injury biomarkers CK and LDH	Khodabukus et al., 2020
		Post deflection	Primary Mouse myoblast	+ 66% (100 ng/ml)	Increase in fiber-CSA (41%)	Vandenburgh et al., 2008
			Immortalized control C57 and mdx myoblast	+ 93% (0.01 µM)		Vandenburgh et al., 2009
			C2C12 myoblast (mouse)	+ 25%	Increase in CF in Dex-induced atrophic tissues (45%*)	Shimizu et al., 2017
			Derived myoblast from human dermal fibroblast	+ 72% (100 ng/ml)	Decrease in CF in non-cryopreserved cells (100 ng/ml, 79%*)	Shimizu et al., 2020
Statin-induced myopathy (Rhabdomyolysis)	Lovastatin	Force transducer	Primary Human myoblast	–75% (2 µM)	Dose-dependent lipid accumulation	Madden et al., 2015
			Primary Human myoblast	–53% (5 µM)	Dose-dependent lipid accumulation	Ananthakumar et al., 2020
		Post deflection	Immortalized human myogenic cells	–75% (2 µM)	Increase in the expression of Atrogin-1 and MuRF-1.	Nagashima et al., 2020
	Cerivastatin	Force transducer	Primary Human myoblast	–50% (50 nM)	Decrease in CF (50 nM, 50%*) Dose-dependent lipid accumulation	Madden et al., 2015
			Primary Human myoblast	–85%	Reduction in myotube diameter Dose-dependent decrease in injury biomarkers CK and LDH	Vandenburgh et al., 1996
			Human Skeletal myoblast	–40% (100 nM)	Decrease in CF Decrease in passive force Myofibers Sarcomere degradation	Zhang et al., 2018
		Post deflection	Primary Human myoblast	–62% (10 nM)	Decrease in CF (10 nM, 62%*) Dose-dependent decrease in myotube width	Alave Reyes-Furrer et al., 2021

*Recalculated data.

As shown in Table 2, the most common drugs used in these models are: dexamethasone, a synthetic glucocorticoid commonly used to induce muscle atrophy (Aguilar-Agon et al., 2021), and insulin growth factor 1 (IGF-1), which activates muscle stem cells (satellite cells, SCs) and regulates anabolic and catabolic pathways in skeletal muscle (Yoshida and Delafontaine, 2020). All these studies have shown that CF decreases or increases in a dose-dependent manner when dexamethasone or IGF-1 are applied, respectively. Authors have also identified changes in myotubes diameter and in the percentage of fibers with striation patterns or the expression of specific biomarkers. Finally, other drugs such as lovastatin (Madden et al., 2015; Afshar et al., 2020; Ananthakumar et al., 2020; Nagashima et al., 2020) and cerivastatin Brady et al., 2008; Madden et al., 2015; Zhang et al., 2018 have been used to generate statin-induced myopathy models in these systems.

## Discussion

Regardless of the experimental platform used or the size of the construct, in vitro muscle constructs present a significantly smaller CF compared to native tissue (0.2–2.9% of native muscle Tw-sF). Gene expression analyses performed on engineered constructs have shown that they are similar to fetal muscles and far removed from adult native muscle (Nagashima et al., 2020). The general conclusion is that engineered tissues are still at an immature stage of development. Indeed, some of the latest experimental approaches consider using co-culture with neural cells to stimulate NMJ formation, muscle innervation (Demestre et al., 2015; Zahavi et al., 2015; Rimington et al., 2021), and tissue maturation.

Among the main experimental platforms, the force transducer studies are the ones that have generated the highest amount of CF data. Moreover, this platform reports the highest CF mean values among the in vitro muscle constructs. Since constructs evaluated by the force transducer platforms are also the ones with the largest muscle mass, a proper normalization is necessary to compare the CF of the engineered muscles between the different platforms. When normalizing CF to total CSA, we found a very high data variability for all the parameters analyzed (twitch, tetanus), both in constructs generated by C2C12 cells and human cells. However, the tetanic-to-twitch ratio presented a low data variability, enabling comparison between different studies. Tetanic-to-Twitch ratio is a parameter independent of the CSA, which indicates that the total CSA used is not adequate for CF data normalization among studies, even those using the same platform.

There are several aspects influencing contractile function that largely differ across studies, that is, assessment time, tissue maturation, contractile area, and length and mass of the muscle construct. For instance, the percentage of myotubes with a cross-striated pattern, which accounts for the muscle maturation level, appears to be higher in muscle constructs assessed by force transducers ( > 95%) compared to µpost (73.52%) and cantilever platforms (12%) (Hinds et al., 2011; Ebrahimi et al., 2021; Al Tanoury et al., 2021). Importantly, none of these variables are normalized by total CSA, and many are rarely reported in the literature. One possibility for future analyses would be to use the effective CSA, considering the contractile area. This can be achieved with immunofluorescence analysis of sarcomeric proteins (Sakar et al., 2012; Juhas and Bursac, 2014). In addition, this analysis could also provide further valuable data such as muscle maturity level (cross-striation) and sarcomere length, which relates to optimal muscle length.

Our comparative analysis across the experimental platforms shows that sF values reported with the post deflection platform are substantially lower than those with force transducers. This may be due to several factors, including a lower contractile area, lower maturation level, and/or a suboptimal sarcomeric length. In the force transducer, the initial length, L_0_, in the resting state before contraction stimulation is adjustable, allowing pre-tension of the construct and maximization of CF. However, it is unattainable to control muscle length in the post deflection method or the cantilever system. In µposts, there is a shortening of L_0_ caused by the passive tension of the construct, that is, a progressive contraction of the cells and hydrogel during maturation. Muscle construct shortening in µposts may vary depending on the elastic modulus and would be more pronounced in hydrogels presenting lower elastic properties, such as fibrin, than collagen (Hinds et al., 2011; Cvetkovic et al., 2014). Due to limited available data, it is not possible to determine to what extent this factor affects the sF readout of µposts experiments. However, data extracted from few selected studies suggest that the sarcomere length does not differ substantially between cantilever deflection (1.58 µm, Santoso et al., 2021), post-deflection (2.76 µm, Ebrahimi et al., 2021) and force transducer platforms (1.5–2.7 µm, Hinds et al., 2011; Rao et al., 2018). Moreover, these data are in line with the optimal sarcomere length (about 2 µm) that produces the maximum tension in native muscles (Moo et al., 2020).

Another factor that may account for the lower sF values in µpost muscle constructs compared to force transducers is the muscle mass. Thus, in the force transducer platform, the muscle construct is undoubtedly longer, about 12–20 mm (Gholobova et al., 2020b) compared to 4 mm, in the case of µposts (Vandenburgh et al., 2008). The total CSA would not fully normalize for longer muscle constructs with similar CSA, where more muscle mass contributes to muscle contraction. Hence, an inappropriate CF normalization may partly account for higher sF values observed on average in force transducer constructs compared to post-deflection.

Finally, an unexpected finding derived from our analysis is the overall higher tetanic-to-twitch ratio values of muscle constructs in µpost (1.5–5.6) compared to constructs assessed by force transducers (1-3). Native rat and human muscles present tetanic-to-twitch ratios in the range of 4–10 (Cheng et al., 2014a), so constructs in µposts appear to recapitulate more closely this feature of native muscles. We hypothesize that constructs assessed using force transducers may suffer undue stress due to the additional manipulation required for FC measurement, which may cause malfunction or fatigue, limiting the tetanic response.

Multiple physiological conditions still need to be adjusted and accounted for in order to achieve higher and more reliable contractile performance data in engineered muscles. In light of the CF and tetanic-to-twitch ratio data, it appears that the use of the ratio would limit the variability of CF data, rendering it a useful parameter for CF data comparison across studies.

## Conclusions and future challenges

CF is a key parameter used to assess the actual functionality of in vitro muscle models. In this review, we have discussed the three main methodologies commonly used to measure CF in muscle in vitro systems: cantilever deflection, post deflection, and force transducers. These platforms cover the evaluation of 2D and 3D in vitro skeletal muscle models, from myotubes to muscle engineered tissue in healthy and disease models.

Currently, there is no standard parameter to express CF for in vitro muscle systems. Thus, we have used specific contractile force (sF), by normalizing either twitch or tetanic contraction with the calculated whole cross-sectional area of the engineered tissue in order to compare all the relevant studies. We believe, however, that a more accurate way to calculate sF would be to use the effective-CSA to normalize CF. This would require analyzing the specific number of myotubes in the CSA, which is unfortunately not available in many studies up to date. Therefore, for future studies, we propose that contractility studies in skeletal muscle constructs should include information related to the construct size (length and diameter), contractile area (i.e. myotube coverage in CSA), maturity level (i.e. % of myotubes showing cross-striations), and sarcomere length to complement the information on contractile performance. In any case, we highly recommend including the tetanic-to-twitch ratio since this parameter provides unique information about muscle contractile performance. Furthermore, our analysis suggests that it may enable proper comparison among different studies. Future studies will help us understand the relative impact of these variables on the force performance of muscle constructs and, consequently, the best methodology for measuring and normalizing CF data.

To date, 3D models have evidenced the highest CF; however sF values present a high variability, with no significant differences among the platforms, and they are still far from the native sF range. There is a certain consensus that the cause of this reduced contractile capacity is a maturation deficit of the tissue constructs. In this line, different approaches are being developed to obtain more physiologically relevant muscle models by implementing vascularization, innervation, and mechanical or electromechanical stimulation. Nevertheless, how these new experimental developments would influence the CF of engineered muscle constructs remains unresolved.

Even though CF is a practical, distinctive parameter for assessing muscle function, it is highly influenced by intrinsic properties of the muscle constructs, so the assessment should be complemented alongside other methodologies, such as immunofluorescence, Ca^2+^ handling, and protein quantification. Future investigations should provide insights into integrating this information with contractile values reported in the literature. Moreover, we expect the emerging CF assessment technologies to provide unique insights into intracellular contractile mechanisms.

Given the growing interest in 3D models and the advances in this field, we anticipate a new generation of in vitro systems that will become the driving force behind subsequent research discoveries. Thus, there are still many opportunities for cooperation in these developments and a need for creative and technological researchers to make it possible.

## References

[bib1] Afshar ME, Abraha HY, Bakooshli MA, Davoudi S, Thavandiran N, Tung K, Ahn H, Ginsberg HJ, Zandstra PW, Gilbert PM (2020). A 96-well culture platform enables longitudinal analyses of engineered human skeletal muscle microtissue strength. Scientific Reports.

[bib2] Afshar Bakooshli M, Lippmann ES, Mulcahy B, Iyer N, Nguyen CT, Tung K, Stewart BA, van den Dorpel H, Fuehrmann T, Shoichet M, Bigot A, Pegoraro E, Ahn H, Ginsberg H, Zhen M, Ashton RS, Gilbert PM (2019). A 3D culture model of innervated human skeletal muscle enables studies of the adult neuromuscular junction. eLife.

[bib3] Agrawal G, Aung A, Varghese S (2017). Skeletal muscle-on-a-chip: an in vitro model to evaluate tissue formation and injury. Lab on a Chip.

[bib4] Aguilar-Agon KW, Capel AJ, Martin NRW, Player DJ, Lewis MP (2019). Mechanical loading stimulates hypertrophy in tissue-engineered skeletal muscle: Molecular and phenotypic responses. Journal of Cellular Physiology.

[bib5] Aguilar-Agon KW, Capel AJ, Fleming JW, Player DJ, Martin NRW, Lewis MP (2021). Mechanical loading of tissue engineered skeletal muscle prevents dexamethasone induced myotube atrophy. Journal of Muscle Research and Cell Motility.

[bib6] Akimoto T, Ushida T, Miyaki S, Tateishi T, Fukubayashi T (2001). Mechanical stretch is a down-regulatory signal for differentiation of C2C12 myogenic cells. Materials Science and Engineering.

[bib7] Akiyama Y, Nakayama A, Nakano S, Amiya R, Hirose J (2021). An Electrical Stimulation Culture System for Daily Maintenance-Free Muscle Tissue Production. Cyborg and Bionic Systems.

[bib8] Al Tanoury Z, Zimmerman JF, Rao J, Sieiro D, McNamara HM, Cherrier T, Rodríguez-delaRosa A, Hick-Colin A, Bousson F, Fugier-Schmucker C, Marchiano F, Habermann B, Chal J, Nesmith AP, Gapon S, Wagner E, Gupta VA, Bassel-Duby R, Olson EN, Cohen AE, Parker KK, Pourquié O (2021). Prednisolone rescues Duchenne muscular dystrophy phenotypes in human pluripotent stem cell-derived skeletal muscle in vitro. PNAS.

[bib9] Alave Reyes-Furrer A, De Andrade S, Bachmann D, Jeker H, Steinmann M, Accart N, Dunbar A, Rausch M, Bono E, Rimann M, Keller H (2021). Matrigel 3D bioprinting of contractile human skeletal muscle models recapitulating exercise and pharmacological responses. Communications Biology.

[bib10] Alcazar J, Csapo R, Ara I, Alegre LM (2019). On the Shape of the Force-Velocity Relationship in Skeletal Muscles: The Linear, the Hyperbolic, and the Double-Hyperbolic. Frontiers in Physiology.

[bib11] Algar WR, Hildebrandt N, Vogel SS, Medintz IL (2019). FRET as a biomolecular research tool - understanding its potential while avoiding pitfalls. Nature Methods.

[bib12] Ananthakumar A, Liu Y, Fernandez CE, Truskey GA, Voora D (2020). Modeling statin myopathy in a human skeletal muscle microphysiological system. PLOS ONE.

[bib13] Anene-Nzelu CG, Peh KY, Fraiszudeen A, Kuan YH, Ng SH, Toh YC, Leo HL, Yu H (2013). Scalable alignment of three-dimensional cellular constructs in a microfluidic chip. Lab on a Chip.

[bib14] Asano T, Ishizua T, Yawo H (2012). Optically controlled contraction of photosensitive skeletal muscle cells. Biotechnology and Bioengineering.

[bib15] Axion Biosystems (2021). HIGH-RES CONTRACTILITY. https://www.axionbiosystems.com/resources/application-note/high-res-contractility.

[bib16] Badu-Mensah A, Guo X, McAleer CW, Rumsey JW, Hickman JJ (2020). Functional skeletal muscle model derived from SOD1-mutant ALS patient iPSCs recapitulates hallmarks of disease progression. Scientific Reports.

[bib17] Bajaj P, Reddy B, Millet L, Wei C, Zorlutuna P, Bao G, Bashir R (2011). Patterning the differentiation of C2C12 skeletal myoblasts. Integrative Biology.

[bib18] Bersini S, Gilardi M, Ugolini GS, Sansoni V, Talò G, Perego S, Zanotti S, Ostano P, Mora M, Soncini M, Vanoni M, Lombardi G, Moretti M (2018). Engineering an Environment for the Study of Fibrosis: A 3D Human Muscle Model with Endothelium Specificity and Endomysium. Cell Reports.

[bib19] Boonen KJM, Langelaan MLP, Polak RB, van der Schaft DWJ, Baaijens FPT, Post MJ (2010). Effects of a combined mechanical stimulation protocol: Value for skeletal muscle tissue engineering. Journal of Biomechanics.

[bib20] Boontheekul T, Hill EE, Kong HJ, Mooney DJ (2007). Regulating Myoblast Phenotype Through Controlled Gel Stiffness and Degradation. Tissue Engineering.

[bib21] Bottinelli R, Reggiani C (2000). Human skeletal muscle fibres: molecular and functional diversity. Progress in Biophysics and Molecular Biology.

[bib22] Brady MA, Lewis MP, Mudera V (2008). Synergy between myogenic and non-myogenic cells in a 3D tissue-engineered craniofacial skeletal muscle construct. Journal of Tissue Engineering and Regenerative Medicine.

[bib23] Bruegmann T, Malan D, Hesse M, Beiert T, Fuegemann CJ, Fleischmann BK, Sasse P (2010). Optogenetic control of heart muscle in vitro and in vivo. Nature Methods.

[bib24] Candiani G, Milano P, Adele S, Dialybrid R, Pharmacell NS (2010). Special Issue “Novel Research about Biomechanics and Biomaterials Used in Hip, Knee and Related Joints.”. Journal of Applied Biomaterials & Biomechanics.

[bib25] Capel AJ, Rimington RP, Fleming JW, Player DJ, Baker LA, Turner MC, Jones JM, Martin NRW, Ferguson RA, Mudera VC, Lewis MP (2019). Scalable 3D Printed Molds for Human Tissue Engineered Skeletal Muscle. Frontiers in Bioengineering and Biotechnology.

[bib26] Cheesbrough A, Sciscione F, Riccio F, Harley P, R’Bibo L, Ziakas G, Darbyshire A, Lieberam I, Song W (2022). Biobased Elastomer Nanofibers Guide Light-Controlled Human-iPSC-Derived Skeletal Myofibers. Advanced Materials (Deerfield Beach, Fla.).

[bib27] Cheng CS, Davis BNJ, Madden L, Bursac N, Truskey GA (2014a). Physiology and metabolism of tissue-engineered skeletal muscle. Experimental Biology and Medicine.

[bib28] Cheng CS, El-Abd Y, Bui K, Hyun YE, Hughes RH, Kraus WE, Truskey GA (2014b). Conditions that promote primary human skeletal myoblast culture and muscle differentiation in vitro. American Journal of Physiology-Cell Physiology.

[bib29] Christensen RK, von Halling Laier C, Kiziltay A, Wilson S, Larsen NB (2020). 3D Printed Hydrogel Multiassay Platforms for Robust Generation of Engineered Contractile Tissues. Biomacromolecules.

[bib30] Cost A-L, Ringer P, Chrostek-Grashoff A (2015). How to Measure Molecular Forces in Cells: A Guide to Evaluating Genetically-Encoded FRET-Based Tension Sensors. Cellular and Molecular Bioengineering.

[bib31] Costantini M, Testa S, Fornetti E, Fuoco C, Sanchez Riera C, Nie M, Bernardini S, Rainer A, Baldi J, Zoccali C, Biagini R, Castagnoli L, Vitiello L, Blaauw B, Seliktar D, Święszkowski W, Garstecki P, Takeuchi S, Cesareni G, Cannata S, Gargioli C (2021). Biofabricating murine and human myo-substitutes for rapid volumetric muscle loss restoration. EMBO Molecular Medicine.

[bib32] Cvetkovic C, Raman R, Chan V, Williams BJ, Tolish M, Bajaj P, Sakar MS, Asada HH, Saif MTA, Bashir R (2014). Three-dimensionally printed biological machines powered by skeletal muscle. PNAS.

[bib33] Demestre M, Orth M, Föhr KJ, Achberger K, Ludolph AC, Liebau S, Boeckers TM (2015). Formation and characterisation of neuromuscular junctions between hiPSC derived motoneurons and myotubes. Stem Cell Research.

[bib34] Denes LT, Riley LA, Mijares JR, Arboleda JD, McKee K, Esser KA, Wang ET (2019). Culturing C2C12 myotubes on micromolded gelatin hydrogels accelerates myotube maturation. Skeletal Muscle.

[bib35] Dennis RG, Kosnik, Ii PE (2000). Excitability and isometric contractile properties of mammalian skeletal muscle constructs engineered in vitroexcitability and isometric contractile properties of mammalian skeletal muscle constructs engineered in vitro. In Vitro Cellular & Developmental Biology - Animal.

[bib36] Dennis RG, Kosnik PE, Gilbert ME, Faulkner JA (2001). Excitability and contractility of skeletal muscle engineered from primary cultures and cell lines. American Journal of Physiology. Cell Physiology.

[bib37] Dhawan V, Lytle IF, Dow DE, Huang YC, Brown DL (2007). Neurotization improves contractile forces of tissue-engineered skeletal muscle. Tissue Engineering.

[bib38] Donnelly K, Khodabukus A, Philp A, Deldicque L, Dennis RG, Baar K (2010). A novel bioreactor for stimulating skeletal muscle in vitro. Tissue Engineering. Part C, Methods.

[bib39] Ebrahimi M, Lad H, Fusto A, Tiper Y, Datye A, Nguyen CT, Jacques E, Moyle LA, Nguyen T, Musgrave B, Chávez-Madero C, Bigot A, Chen C, Turner S, Stewart BA, Pegoraro E, Vitiello L, Gilbert PM (2021). De novo revertant fiber formation and therapy testing in a 3D culture model of Duchenne muscular dystrophy skeletal muscle. Acta Biomaterialia.

[bib40] Fernández-Costa JM, Fernández-Garibay X, Velasco-Mallorquí F, Ramón-Azcón J (2021). Bioengineered *in vitro* skeletal muscles as new tools for muscular dystrophies preclinical studies. Journal of Tissue Engineering.

[bib41] Fernández-Garibay X, Ortega MA, Cerro-Herreros E, Comelles J, Martínez E, Artero R, Fernández-Costa JM, Ramón-Azcón J (2021). Bioengineeredin vitro3D model of myotonic dystrophy type 1 human skeletal muscle. Biofabrication.

[bib42] Fitts RH, McDonald KS, Schluter JM (1991). The determinants of skeletal muscle force and power: their adaptability with changes in activity pattern. Journal of Biomechanics.

[bib43] Fujita H, Nedachi T, Kanzaki M (2007). Accelerated de novo sarcomere assembly by electric pulse stimulation in C2C12 myotubes. Experimental Cell Research.

[bib44] Fujita H, Shimizu K, Nagamori E (2009). Novel method for fabrication of skeletal muscle construct from the C2C12 myoblast cell line using serum-free medium AIM-V. Biotechnology and Bioengineering.

[bib45] Fujita H, Dau VT, Shimizu K, Hatsuda R, Sugiyama S, Nagamori E (2010). Designing of a Si-MEMS device with an integrated skeletal muscle cell-based bio-actuator. Biomedical Microdevices.

[bib46] Furuhashi M, Morimoto Y, Shima A, Nakamura F, Ishikawa H, Takeuchi S (2021). Formation of contractile 3D bovine muscle tissue for construction of millimetre-thick cultured steak. Npj Science of Food.

[bib47] Gao L, Akhtar MU, Yang F, Ahmad S, He J, Lian Q, Cheng W, Zhang J, Li D (2021). Recent progress in engineering functional biohybrid robots actuated by living cells. Acta Biomaterialia.

[bib48] Gates EM, LaCroix AS, Rothenberg KE, Hoffman BD (2019). Improving Quality, Reproducibility, and Usability of FRET-Based Tension Sensors. Cytometry. Part A.

[bib49] Gholobova D, Terrie L, Gerard M, Declercq H, Thorrez L (2020a). Vascularization of tissue-engineered skeletal muscle constructs. Biomaterials.

[bib50] Gholobova D, Terrie L, Mackova K, Desender L, Carpentier G, Gerard M, Hympanova L, Deprest J, Thorrez L (2020b). Functional evaluation of prevascularization in one-stage versus two-stage tissue engineering approach of human bio-artificial muscle. Biofabrication.

[bib51] Gilbert-Honick J, Iyer SR, Somers SM, Lovering RM, Wagner K, Mao HQ, Grayson WL (2018). Engineering functional and histological regeneration of vascularized skeletal muscle. Biomaterials.

[bib52] Gilbert-Honick J, Iyer SR, Somers SM, Takasuka H, Lovering RM, Wagner KR, Mao HQ, Grayson WL (2020). Engineering 3D skeletal muscle primed for neuromuscular regeneration following volumetric muscle loss. Biomaterials.

[bib53] Grashoff C, Hoffman BD, Brenner MD, Zhou R, Parsons M, Yang MT, McLean MA, Sligar SG, Chen CS, Ha T, Schwartz MA (2010). Measuring mechanical tension across vinculin reveals regulation of focal adhesion dynamics. Nature.

[bib54] Grassi B, Rossiter HB, Zoladz JA (2015). Skeletal muscle fatigue and decreased efficiency: two sides of the same coin?. Exercise and Sport Sciences Reviews.

[bib55] Gunther LK, Rohde JA, Tang W, Cirilo JA, Marang CP, Scott BD, Thomas DD, Debold EP, Yengo CM (2020). FRET and optical trapping reveal mechanisms of actin activation of the power stroke and phosphate release in myosin V. The Journal of Biological Chemistry.

[bib56] Guo J, Wang Y, Sachs F, Meng F (2014). Actin stress in cell reprogramming. PNAS.

[bib57] Guo X, Badu-Mensah A, Thomas MC, McAleer CW, Hickman JJ (2020). Characterization of Functional Human Skeletal Myotubes and Neuromuscular Junction Derived-From the Same Induced Pluripotent Stem Cell Source. Bioengineering (Basel, Switzerland).

[bib58] Gupta D, Santoso JW, McCain ML (2021). Characterization of Gelatin Hydrogels Cross-Linked with Microbial Transglutaminase as Engineered Skeletal Muscle Substrates. Bioengineering (Basel, Switzerland).

[bib59] Hansen A, Eder A, Bönstrup M, Flato M, Mewe M, Schaaf S, Aksehirlioglu B, Schwoerer AP, Schwörer A, Uebeler J, Eschenhagen T (2010). Development of a drug screening platform based on engineered heart tissue. Circulation Research.

[bib60] Hill C, Brunello E, Fusi L, Ovejero JG, Irving M (2021). Myosin-based regulation of twitch and tetanic contractions in mammalian skeletal muscle. eLife.

[bib61] Hinds S, Bian W, Dennis RG, Bursac N (2011). The role of extracellular matrix composition in structure and function of bioengineered skeletal muscle. Biomaterials.

[bib62] Hofemeier AD, Limon T, Muenker TM, Wallmeyer B, Jurado A, Afshar ME, Ebrahimi M, Tsukanov R, Oleksiievets N, Enderlein J, Gilbert PM, Betz T (2021). Global and local tension measurements in biomimetic skeletal muscle tissues reveals early mechanical homeostasis. eLife.

[bib63] Horvath P, Aulner N, Bickle M, Davies AM, Nery ED, Ebner D, Montoya MC, Östling P, Pietiäinen V, Price LS, Shorte SL, Turcatti G, von Schantz C, Carragher NO (2016). Screening out irrelevant cell-based models of disease. Nature Reviews. Drug Discovery.

[bib64] Huang Y-C, Dennis RG, Larkin L, Baar K (2005). Rapid formation of functional muscle in vitro using fibrin gels. Journal of Applied Physiology (Bethesda, Md.

[bib65] Ikeda K, Ito A, Sato M, Kawabe Y, Kamihira M (2016). Improved contractile force generation of tissue-engineered skeletal muscle constructs by IGF-I and Bcl-2 gene transfer with electrical pulse stimulation. Regenerative Therapy.

[bib66] Ikeda K, Ito A, Imada R, Sato M, Kawabe Y, Kamihira M (2017). In vitro drug testing based on contractile activity of C2C12 cells in an epigenetic drug model. Scientific Reports.

[bib67] Jeon Y, Choi J, Kim HJ, Lee H, Lim JY, Choi SJ (2019). Sex- and fiber-type-related contractile properties in human single muscle fiber. Journal of Exercise Rehabilitation.

[bib68] Juhas M, Bursac N (2014). Roles of adherent myogenic cells and dynamic culture in engineered muscle function and maintenance of satellite cells. Biomaterials.

[bib69] Juhas M, Ye J, Bursac N (2016). Design, evaluation, and application of engineered skeletal muscle. Methods.

[bib70] Juhas M, Abutaleb N, Wang JT, Ye J, Shaikh Z, Sriworarat C, Qian Y, Bursac N (2018). Incorporation of macrophages into engineered skeletal muscle enables enhanced muscle regeneration. Nature Biomedical Engineering.

[bib71] Kaji H, Ishibashi T, Nagamine K, Kanzaki M, Nishizawa M (2010). Electrically induced contraction of C2C12 myotubes cultured on a porous membrane-based substrate with muscle tissue-like stiffness. Biomaterials.

[bib72] Kaur A, Dhakal S (2020). Recent applications of FRET-based multiplexed techniques. TrAC Trends in Analytical Chemistry.

[bib73] Khodabukus A, Baar K (2012). Defined electrical stimulation emphasizing excitability for the development and testing of engineered skeletal muscle. Tissue Engineering. Part C, Methods.

[bib74] Khodabukus A, Baar K (2016). Factors That Affect Tissue-Engineered Skeletal Muscle Function and Physiology. Cells Tissues Organs.

[bib75] Khodabukus A, Madden L, Prabhu NK, Koves TR, Jackman CP, Muoio DM, Bursac N (2019). Electrical stimulation increases hypertrophy and metabolic flux in tissue-engineered human skeletal muscle. Biomaterials.

[bib76] Khodabukus A, Kaza A, Wang J, Prabhu N, Goldstein R, Vaidya VS, Bursac N (2020). Tissue-Engineered Human Myobundle System as a Platform for Evaluation of Skeletal Muscle Injury Biomarkers. Toxicological Sciences.

[bib77] Khodabukus A (2021). Tissue-Engineered Skeletal Muscle Models to Study Muscle Function, Plasticity, and Disease. Frontiers in Physiology.

[bib78] Kim JH, Seol YJ, Ko IK, Kang HW, Lee YK, Yoo JJ, Atala A, Lee SJ (2018). 3D Bioprinted Human Skeletal Muscle Constructs for Muscle Function Restoration. Scientific Reports.

[bib79] Kim H, Kim MC, Asada HH (2019). Extracellular matrix remodelling induced by alternating electrical and mechanical stimulations increases the contraction of engineered skeletal muscle tissues. Scientific Reports.

[bib80] King AM, Loiselle DS, Kohl P (2004). Force Generation for Locomotion of Vertebrates: Skeletal Muscle Overview. IEEE Journal of Oceanic Engineering.

[bib81] Komatsubara AT, Matsuda M, Aoki K (2015). Quantitative analysis of recombination between YFP and CFP genes of FRET biosensors introduced by lentiviral or retroviral gene transfer. Scientific Reports.

[bib82] Kornegay JN (2017). The golden retriever model of Duchenne muscular dystrophy. Skeletal Muscle.

[bib83] Krivickas LS, Dorer DJ, Ochala J, Frontera WR (2011). Relationship between force and size in human single muscle fibres. Experimental Physiology.

[bib84] Lafoux A, Lotteau S, Huchet C, Ducreux S (2020). The Contractile Phenotype of Skeletal Muscle in TRPV1 Knockout Mice is Gender-Specific and Exercise-Dependent. Life (Basel, Switzerland).

[bib85] Langelaan MLP, Boonen KJM, Rosaria-Chak KY, van der Schaft DWJ, Post MJ, Baaijens FPT (2011). Advanced maturation by electrical stimulation: Differences in response between C2C12 and primary muscle progenitor cells. Journal of Tissue Engineering and Regenerative Medicine.

[bib86] Langhammer CG, Zahn JD, Firestein BL (2010). Identification and quantification of skeletal myotube contraction and association in vitro by video microscopy. Cytoskeleton (Hoboken, N.J.).

[bib87] Langhammer CG, Kutzing MK, Luo V, Zahn JD, Firestein BL (2013). A topographically modified substrate-embedded MEA for directed myotube formation at electrode contact sites. Annals of Biomedical Engineering.

[bib88] Larkin LM, Van der Meulen JH, Dennis RG, Kennedy JB (2006). Functional evaluation of nerve-skeletal muscle constructs engineered in vitro. In Vitro Cellular & Developmental Biology. Animal.

[bib89] Lasa-Fernandez H, Mosqueira-Martín L, Alzualde A, Lasa-Elgarresta J, Vallejo-Illarramendi A (2020). A genotyping method combining primer competition PCR with HRM analysis to identify point mutations in Duchenne animal models. Scientific Reports.

[bib90] Lemke SB, Schnorrer F (2017). Mechanical forces during muscle development. Mechanisms of Development.

[bib91] Levenberg S, Rouwkema J, Macdonald M, Garfein ES, Kohane DS, Darland DC, Marini R, van Blitterswijk CA, Mulligan RC, D’Amore PA, Langer R (2005). Engineering vascularized skeletal muscle tissue. Nature Biotechnology.

[bib92] Li B, Lin M, Tang Y, Wang B, Wang JHC (2008). A novel functional assessment of the differentiation of micropatterned muscle cells. Journal of Biomechanics.

[bib93] Lindstedt SL, Lindstedt SL, Hoppeler HH (2016). Skeletal muscle tissue in movement and health: positives and negatives. The Journal of Experimental Biology.

[bib94] Madden L, Juhas M, Kraus WE, Truskey GA, Bursac N (2015). Bioengineered human myobundles mimic clinical responses of skeletal muscle to drugs. eLife.

[bib95] Maffioletti SM, Sarcar S, Henderson ABH, Mannhardt I, Pinton L, Moyle LA, Steele-Stallard H, Cappellari O, Wells KE, Ferrari G, Mitchell JS, Tyzack GE, Kotiadis VN, Khedr M, Ragazzi M, Wang W, Duchen MR, Patani R, Zammit PS, Wells DJ, Eschenhagen T, Tedesco FS (2018). Three-Dimensional Human iPSC-Derived Artificial Skeletal Muscles Model Muscular Dystrophies and Enable Multilineage Tissue Engineering. Cell Reports.

[bib96] Maganaris CN, Baltzopoulos V, Ball D, Sargeant AJ (2001). In vivo specific tension of human skeletal muscle. Journal of Applied Physiology (Bethesda, Md.

[bib97] Mahmoudi P, Veladi H, Pakdel FG (2017). Optogenetics, Tools and Applications in Neurobiology. Journal of Medical Signals and Sensors.

[bib98] Martin NRW, Turner MC, Farrington R, Player DJ, Lewis MP (2017). Leucine elicits myotube hypertrophy and enhances maximal contractile force in tissue engineered skeletal muscle in vitro. Journal of Cellular Physiology.

[bib99] Massobrio P, Tessadori J, Chiappalone M, Ghirardi M (2015). *In Vitro* Studies of Neuronal Networks and Synaptic Plasticity in Invertebrates and in Mammals Using Multielectrode Arrays. Neural Plasticity.

[bib100] McAleer CW, Smith AST, Najjar S, Pirozzi K, Long CJ, Hickman JJ (2014). Mechanistic investigation of adult myotube response to exercise and drug treatment in vitro using a multiplexed functional assay system. Journal of Applied Physiology.

[bib101] Merrill DR, Bikson M, Jefferys JGR (2005). Electrical stimulation of excitable tissue: design of efficacious and safe protocols. Journal of Neuroscience Methods.

[bib102] Miller MS, Bedrin NG, Ades PA, Palmer BM, Toth MJ (2015). Molecular determinants of force production in human skeletal muscle fibers: effects of myosin isoform expression and cross-sectional area. American Journal of Physiology-Cell Physiology.

[bib103] Mills RJ, Parker BL, Monnot P, Needham EJ, Vivien CJ, Ferguson C, Parton RG, James DE, Porrello ER, Hudson JE (2019). Development of a human skeletal micro muscle platform with pacing capabilities. Biomaterials.

[bib104] Moo EK, Leonard TR, Herzog W (2020). The sarcomere force-length relationship in an intact muscle-tendon unit. The Journal of Experimental Biology.

[bib105] Moon DG, Christ G, Stitzel JD, Atala A, Yoo JJ (2008). Cyclic mechanical preconditioning improves engineered muscle contraction. Tissue Engineering. Part A.

[bib106] Nagamine K, Kawashima T, Sekine S, Ido Y, Kanzaki M, Nishizawa M (2011). Spatiotemporally controlled contraction of micropatterned skeletal muscle cells on a hydrogel sheet. Lab on a Chip.

[bib107] Nagashima T, Hadiwidjaja S, Ohsumi S, Murata A, Hisada T, Kato R, Okada Y, Honda H, Shimizu K (2020). In Vitro Model of Human Skeletal Muscle Tissues with Contractility Fabricated by Immortalized Human Myogenic Cells. Advanced Biosystems.

[bib108] Nakamura T, Takagi S, Kamon T, Yamasaki K-I, Fujisato T (2017). Development and evaluation of a removable tissue-engineered muscle with artificial tendons. Journal of Bioscience and Bioengineering.

[bib109] Neal D, Sakar MS, Bashir R, Chan V, Asada HH (2015). Mechanical Characterization and Shape Optimization of Fascicle-Like 3D Skeletal Muscle Tissues Contracted with Electrical and Optical Stimuli. Tissue Engineering. Part A.

[bib110] Nesmith AP, Wagner MA, Pasqualini FS, O’Connor BB, Pincus MJ, August PR, Parker KK (2016). A human in vitro model of Duchenne muscular dystrophy muscle formation and contractility. The Journal of Cell Biology.

[bib111] Nikolić N, Görgens SW, Thoresen GH, Aas V, Eckel J, Eckardt K (2017). Electrical pulse stimulation of cultured skeletal muscle cells as a model for in vitro exercise - possibilities and limitations. Acta Physiologica (Oxford, England).

[bib112] Okano T, Matsuda T (1998). Tissue engineered skeletal muscle: preparation of highly dense, highly oriented hybrid muscular tissues. Cell Transplantation.

[bib113] Osaki T, Sivathanu V, Kamm RD (2018a). Crosstalk between developing vasculature and optogenetically engineered skeletal muscle improves muscle contraction and angiogenesis. Biomaterials.

[bib114] Osaki T, Uzel SGM, Kamm RD (2018b). Microphysiological 3D model of amyotrophic lateral sclerosis (ALS) from human iPS-derived muscle cells and optogenetic motor neurons. Science Advances.

[bib115] Park H, Bhalla R, Saigal R, Radisic M, Watson N, Langer R, Vunjak-Novakovic G (2008). Effects of electrical stimulation in C2C12 muscle constructs. Journal of Tissue Engineering and Regenerative Medicine.

[bib116] Pasquarelli A (2021). Microelectrode Arrays, Implants, and Organs-on-a-chip.

[bib117] Peper S, Vo T, Ahuja N, Awad K, Mikos AG, Varanasi V, Brotto M, Duque G, Invernizzi M, Nader G (2021). Bioprinted nanocomposite hydrogels: A proposed approach to functional restoration of skeletal muscle and vascular tissue following volumetric muscle loss. Current Opinion in Pharmacology.

[bib118] Perez-Puyana V, Wieringa P, Yuste Y, de la Portilla F, Guererro A, Romero A, Moroni L (2021). Fabrication of hybrid scaffolds obtained from combinations of PCL with gelatin or collagen via electrospinning for skeletal muscle tissue engineering. Journal of Biomedical Materials Research. Part A.

[bib119] Pirozzi KL, Long CJ, McAleer CW, Smith AST, Hickman JJ (2013). Correlation of embryonic skeletal muscle myotube physical characteristics with contractile force generation on an atomic force microscope-based bio-microelectromechanical systems device. Applied Physics Letters.

[bib120] Pollot BE, Rathbone CR, Wenke JC, Guda T (2018). Natural polymeric hydrogel evaluation for skeletal muscle tissue engineering. Journal of Biomedical Materials Research. Part B, Applied Biomaterials.

[bib121] Powell C, Shansky J, Del Tatto M, Forman DE, Hennessey J, Sullivan K, Zielinski BA, Vandenburgh HH (1999). Tissue-engineered human bioartificial muscles expressing a foreign recombinant protein for gene therapy. Human Gene Therapy.

[bib122] Powell CA, Smiley BL, Mills J, Vandenburgh HH (2002). Mechanical stimulation improves tissue-engineered human skeletal muscle. American Journal of Physiology. Cell Physiology.

[bib123] Qazi TH, Mooney DJ, Pumberger M, Geissler S, Duda GN (2015). Biomaterials based strategies for skeletal muscle tissue engineering: existing technologies and future trends. Biomaterials.

[bib124] Rabieh N, Ojovan SM, Shmoel N, Erez H, Maydan E, Spira ME (2016). On-chip, multisite extracellular and intracellular recordings from primary cultured skeletal myotubes. Scientific Reports.

[bib125] Rangarajan S, Madden L, Bursac N (2014). Use of flow, electrical, and mechanical stimulation to promote engineering of striated muscles. Annals of Biomedical Engineering.

[bib126] Rao L, Qian Y, Khodabukus A, Ribar T, Bursac N (2018). Engineering human pluripotent stem cells into a functional skeletal muscle tissue. Nature Communications.

[bib127] Rausch M, Böhringer D, Steinmann M, Schubert DW, Schrüfer S, Mark C, Fabry B (2020). Measurement of Skeletal Muscle Fiber Contractility with High-Speed Traction Microscopy. Biophysical Journal.

[bib128] Raymackers JM, Debaix H, Colson-Van Schoor M, De Backer F, Tajeddine N, Schwaller B, Gailly P, Gillis JM (2003). Consequence of parvalbumin deficiency in the mdx mouse: histological, biochemical and mechanical phenotype of a new double mutant. Neuromuscular Disorders.

[bib129] Ribeiro AJS, Denisin AK, Wilson RE, Pruitt BL (2016). For whom the cells pull: Hydrogel and micropost devices for measuring traction forces. Methods (San Diego, Calif.).

[bib130] Rimington RP, Fleming JW, Capel AJ, Wheeler PC, Lewis MP (2021). Bioengineered model of the human motor unit with physiologically functional neuromuscular junctions. Scientific Reports.

[bib131] Ruedinger F, Lavrentieva A, Blume C, Pepelanova I, Scheper T (2015). Hydrogels for 3D mammalian cell culture: a starting guide for laboratory practice. Applied Microbiology and Biotechnology.

[bib132] Ruzgys P, Jakutavičiūtė M, Šatkauskienė I, Čepurnienė K, Šatkauskas S (2019). Effect of electroporation medium conductivity on exogenous molecule transfer to cells in vitro. Scientific Reports.

[bib133] Sakar MS, Neal D, Boudou T, Borochin MA, Li Y, Weiss R, Kamm RD, Chen CS, Asada HH (2012). Formation and optogenetic control of engineered 3D skeletal muscle bioactuators. Lab on a Chip.

[bib134] Sala L, van Meer BJ, Tertoolen LGJ, Bakkers J, Bellin M, Davis RP, Denning C, Dieben MAE, Eschenhagen T, Giacomelli E, Grandela C, Hansen A, Holman ER, Jongbloed MRM, Kamel SM, Koopman CD, Lachaud Q, Mannhardt I, Mol MPH, Mosqueira D, Orlova VV, Passier R, Ribeiro MC, Saleem U, Smith GL, Burton FL, Mummery CL (2018). MUSCLEMOTION. Circulation Research.

[bib135] Salgado-Almario J, Vicente M, Molina Y, Martinez-Sielva A, Vincent P, Domingo B, Llopis J (2022). Simultaneous imaging of calcium and contraction in the beating heart of zebrafish larvae. Theranostics.

[bib136] Santoso JW, Li X, Gupta D, Suh GC, Hendricks E, Lin S, Perry S, Ichida JK, Dickman D, McCain ML (2021). Engineering skeletal muscle tissues with advanced maturity improves synapse formation with human induced pluripotent stem cell-derived motor neurons. APL Bioengineering.

[bib137] Sato M, Ito A, Akiyama H, Kawabe Y, Kamihira M (2013). Effects of B-cell lymphoma 2 gene transfer to myoblast cells on skeletal muscle tissue formation using magnetic force-based tissue engineering. Tissue Engineering. Part A.

[bib138] Shadrin IY, Khodabukus A, Bursac N (2016). Striated muscle function, regeneration, and repair. Cellular and Molecular Life Sciences.

[bib139] Shimizu K, Sasaki H, Hida H, Fujita H, Obinata K, Shikida M, Nagamori E (2010). Assembly of skeletal muscle cells on a Si-MEMS device and their generative force measurement. Biomedical Microdevices.

[bib140] Shimizu K, Araki H, Sakata K, Tonomura W, Hashida M, Konishi S (2015). Microfluidic devices for construction of contractile skeletal muscle microtissues. Journal of Bioscience and Bioengineering.

[bib141] Shimizu K, Genma R, Gotou Y, Nagasaka S, Honda H (2017). Three-Dimensional Culture Model of Skeletal Muscle Tissue with Atrophy Induced by Dexamethasone. Bioengineering.

[bib142] Shimizu K, Ohsumi S, Kishida T, Mazda O, Honda H (2020). Fabrication of contractile skeletal muscle tissues using directly converted myoblasts from human fibroblasts. Journal of Bioscience and Bioengineering.

[bib143] Shrestha D, Jenei A, Nagy P, Vereb G, Szöllősi J (2015). Understanding FRET as a research tool for cellular studies. International Journal of Molecular Sciences.

[bib144] Smith AST, Long CJ, Pirozzi K, Najjar S, McAleer C, Vandenburgh HH, Hickman JJ (2014). A multiplexed chip-based assay system for investigating the functional development of human skeletal myotubes in vitro. Journal of Biotechnology.

[bib145] Smith AST, Davis J, Lee G, Mack DL, Kim DH (2016). Muscular dystrophy in a dish: engineered human skeletal muscle mimetics for disease modeling and drug discovery. Drug Discovery Today.

[bib146] Sun Y, Duffy R, Lee A, Feinberg AW (2013). Optimizing the structure and contractility of engineered skeletal muscle thin films. Acta Biomaterialia.

[bib147] Takagi S, Nakamura T, Fujisato T (2018). Effect of heat stress on contractility of tissue-engineered artificial skeletal muscle. Journal of Artificial Organs.

[bib148] Takahashi H, Shimizu T, Okano T (2018). Engineered Human Contractile Myofiber Sheets as a Platform for Studies of Skeletal Muscle Physiology. Scientific Reports.

[bib149] Thorrez L, DiSano K, Shansky J, Vandenburgh H (2018). Engineering of Human Skeletal Muscle With an Autologous Deposited Extracellular Matrix. Frontiers in Physiology.

[bib150] Toral-Ojeda Ivan, Aldanondo G, Lasa-Elgarresta J, Lasa-Fernández H, Fernández-Torrón R, López de Munain A, Vallejo-Illarramendi A (2016). Calpain 3 deficiency affects SERCA expression and function in the skeletal muscle. Expert Reviews in Molecular Medicine.

[bib151] Toral-Ojeda I, Aldanondo G, Lasa-Elgarresta J, Lasa-Fernandez H, Vesga-Castro C, Mouly V, Munain A de, Vallejo-Illarramendi A (2018). A Novel Functional In Vitro Model that Recapitulates Human Muscle Disorders.

[bib152] Uchimura T, Asano T, Nakata T, Hotta A, Sakurai H (2021). A muscle fatigue-like contractile decline was recapitulated using skeletal myotubes from Duchenne muscular dystrophy patient-derived iPSCs. Cell Reports. Medicine.

[bib153] Urbanchek MG, Picken EB, Kalliainen LK, Kuzon WM (2001). Specific force deficit in skeletal muscles of old rats is partially explained by the existence of denervated muscle fibers. The Journals of Gerontology. Series A, Biological Sciences and Medical Sciences.

[bib154] Urciuolo A, Serena E, Ghua R, Zatti S, Giomo M, Mattei N, Vetralla M, Selmin G, Luni C, Vitulo N, Valle G, Vitiello L, Elvassore N, Asakura A (2020). Engineering a 3D in vitro model of human skeletal muscle at the single fiber scale. PLOS ONE.

[bib155] Vallejo-Illarramendi A, Toral-Ojeda I, Aldanondo G, López de Munain A (2014). Dysregulation of calcium homeostasis in muscular dystrophies. Expert Reviews in Molecular Medicine.

[bib156] van der Schaft DWJ, van Spreeuwel ACC, van Assen HC, Baaijens FPT (2011). Mechanoregulation of Vascularization in Aligned Tissue-Engineered Muscle: A Role for Vascular Endothelial Growth Factor. Tissue Engineering Part A.

[bib157] Vandenburgh HH, Karlisch P (1989). Longitudinal growth of skeletal myotubes in vitro in a new horizontal mechanical cell stimulator. In Vitro Cellular & Developmental Biology.

[bib158] Vandenburgh HH, Hatfaludy S, Karlisch P, Shansky J (1991). Mechanically induced alterations in cultured skeletal muscle growth. Journal of Biomechanics.

[bib159] Vandenburgh H, Del Tatto M, Shansky J, Lemaire J, Chang A, Payumo F, Lee P, Goodyear A, Raven L (1996). Tissue-engineered skeletal muscle organoids for reversible gene therapy. Human Gene Therapy.

[bib160] Vandenburgh H, Shansky J, Benesch-Lee F, Barbata V, Reid J, Thorrez L, Valentini R, Crawford G (2008). Drug-screening platform based on the contractility of tissue-engineered muscle. Muscle & Nerve.

[bib161] Vandenburgh H, Shansky J, Benesch-Lee F, Skelly K, Spinazzola JM, Saponjian Y, Tseng BS (2009). Automated drug screening with contractile muscle tissue engineered from dystrophic myoblasts. FASEB Journal.

[bib162] Vandenburgh H (2010). High-content drug screening with engineered musculoskeletal tissues. Tissue Engineering. Part B, Reviews.

[bib163] Vila OF, Chavez M, Ma SP, Yeager K, Zholudeva LV, Colón-Mercado JM, Qu Y, Nash TR, Lai C, Feliciano CM, Carter M, Kamm RD, Judge LM, Conklin BR, Ward ME, McDevitt TC, Vunjak-Novakovic G (2021). Bioengineered optogenetic model of human neuromuscular junction. Biomaterials.

[bib164] Wan L, Flegle J, Ozdoganlar B, LeDuc PR (2020). Toward Vasculature in Skeletal Muscle-on-a-Chip through Thermo-Responsive Sacrificial Templates. Micromachines.

[bib165] Wang J, Khodabukus A, Rao L, Vandusen K, Abutaleb N, Bursac N (2019). Engineered skeletal muscles for disease modeling and drug discovery. Biomaterials.

[bib166] Widmaier E, Raff H (2008). Human Physiology - The Mechanisms of Body Function.

[bib167] Wilson K, Molnar P, Hickman J (2007). Integration of functional myotubes with a Bio-MEMS device for non-invasive interrogation. Lab on a Chip.

[bib168] Wilson K, Das M, Wahl KJ, Colton RJ, Hickman J (2010). Measurement of contractile stress generated by cultured rat muscle on silicon cantilevers for toxin detection and muscle performance enhancement. PLOS ONE.

[bib169] Wu L, Huang C, Emery BP, Sedgwick AC, Bull SD, He XP, Tian H, Yoon J, Sessler JL, James TD (2020). Förster resonance energy transfer (FRET)-based small-molecule sensors and imaging agents. Chemical Society Reviews.

[bib170] Xu B, Zhang M, Perlingeiro RCR, Shen W (2019). Skeletal Muscle Constructs Engineered from Human Embryonic Stem Cell Derived Myogenic Progenitors Exhibit Enhanced Contractile Forces When Differentiated in a Medium Containing EGM-2 Supplements. Advanced Biosystems.

[bib171] Yamamoto Y, Ito A, Kato M, Kawabe Y, Shimizu K, Fujita H, Nagamori E, Kamihira M (2009). Preparation of artificial skeletal muscle tissues by a magnetic force-based tissue engineering technique. Journal of Bioscience and Bioengineering.

[bib172] Yamamoto Y, Ito A, Fujita H, Nagamori E, Kawabe Y, Kamihira M (2011). Functional Evaluation of Artificial Skeletal Muscle Tissue Constructs Fabricated by a Magnetic Force-Based Tissue Engineering Technique. Tissue Engineering Part A.

[bib173] Yoshida T, Delafontaine P (2020). Mechanisms of IGF-1-Mediated Regulation of Skeletal Muscle Hypertrophy and Atrophy. Cells.

[bib174] Yoshioka K, Ito A, Kawabe Y, Kamihira M (2020). Novel neuromuscular junction model in 2D and 3D myotubes co-cultured with induced pluripotent stem cell-derived motor neurons. Journal of Bioscience and Bioengineering.

[bib175] Yoshioka K, Ito A, Arifuzzaman M, Yoshigai T, Fan F, Sato K, Shimizu K, Kawabe Y, Kamihira M (2021a). Miniaturized skeletal muscle tissue fabrication for measuring contractile activity. Journal of Bioscience and Bioengineering.

[bib176] Yoshioka K, Ito A, Horie M, Ikeda K, Kataoka S, Sato K, Yoshigai T, Sakurai H, Hotta A, Kawabe Y, Kamihira M, Tremblay JP (2021b). Contractile Activity of Myotubes Derived from Human Induced Pluripotent Stem Cells: A Model of Duchenne Muscular Dystrophy. Cells.

[bib177] Yusuf F, Brand-Saberi B (2012). Myogenesis and muscle regeneration. Histochemistry and Cell Biology.

[bib178] Zahavi EE, Ionescu A, Gluska S, Gradus T, Ben-Yaakov K, Perlson E (2015). A compartmentalized microfluidic neuromuscular co-culture system reveals spatial aspects of GDNF functions. Journal of Cell Science.

[bib179] Zhang F, Wang LP, Boyden ES, Deisseroth K (2006). Channelrhodopsin-2 and optical control of excitable cells. Nature Methods.

[bib180] Zhang X, Hong S, Yen R, Kondash M, Fernandez CE, Truskey GA (2018). A system to monitor statin-induced myopathy in individual engineered skeletal muscle myobundles. Lab on a Chip.

[bib181] Zhuang P, An J, Chua CK, Tan LP (2020). Bioprinting of 3D in vitro skeletal muscle models: A review. Materials & Design.

