# Supplementary material for: Contractile force assessment methods for in vitro skeletal muscle tissues
Source: eLife. 2022 May 23;11:e77204. doi: 10.7554/eLife.77204 (PMC9126583; doi:10.7554/eLife.77204)
Supplement: Table 1—source data 1. [file elife-77204-table1-data1.pdf]

Table S1. Complementary information to Table 1. Includes additional information specific to the experimental setup and stimulation parameters.

	CELL SOURCE	EXPERIMENTAL SETUP	EVALUATION TIME	STIMULATION	TWITCH CONTRACTIONS		TETANUS CONTRACTION		REFERENCE
					STIMULUS	CF	STIMULUS	CF	
**CANTILEVER DEFLECTION**	C2C12 myoblasts (mouse)	Laminin/cell-tak coated Si-cantilevers<br>Spring constant (K) = 0.327 N/m	Day 7	Electrical	0.64 V/mm<br>1-3 Hz	0.54 ± 0.02 µN (1Hz)<br>0.45 ± 0.03 µN (2Hz)<br>0.26 ± 0.01 µN (3Hz)	0.64 V/mm<br>20 Hz<br>For 4 s	1.01 ± 0.14 µN	*Shimizu et al., 2010*
	Rat myoblasts (embryonic)	DETA-coated cantilevers<br>(755 µm x 109 µm x 3 µm)<br>Spring constant (k) = 1.27 ± 0.06 N/m	Day 10-13	Electrical	5 V DC<br>1 Hz	1.3 kPa	—	—	*Wilson et al., 2010*
	C2C12 myoblasts (mouse)	Fibronectin-patterning MTF	Day 6	Electrical	1 Hz<br>10 ms	9.4 ± 4.6 kPa	20 Hz<br>10 ms	—	*Sun et al., 2013*
	Rat myoblasts (embryonic)	DETA-coated cantilevers<br>(750 µm x 100 µm x 4 µm) in a 35 mm-stimulation chamber	Day 12 - 14	Electrical	5 V DC<br>40 ms<br>1 Hz	38 - 256 nN<br>(Stress. 350 - 1760 Pa)<br>Stoney's Eq.<br>25 - 181 nN<br>(Stress. 168 - 1170 Pa)<br>FEA	—	—	*Pirozzi et al., 2013*
	Primary human myoblast	DETA-coated microscale silicon cantilevers on a heated culture dish	Day 14 - 21	Electrical	3 V<br>40 ms<br>1 Hz	32 nN (Day 16)[g]<br>140 nN (Day 23)[g]	—	—	*Smith et al., 2014*
	Rat myoblasts (adult)	DETA-coated microscale silicon cantilevers on a heated culture dish	Day 4 - 7	Electrical<br>Biochemical	3 V<br>40 ms<br>1 Hz	24.13 nN (Control)g<br>76.92 nN (Creatine)g<br>169.68 nN (CLFS)g	—	—	*McAleer et al., 2014*
	Human myoblasts	PDMS thin film	Day 3 - 6	Electrical	10 V<br>1-23 Hz	Healthy<br>3.46 kPa (Day 3)[g]<br>9.97 kPa (Day 6)[g]	—	—	*Nesmith et al., 2016*
	Human induced pluripotent stem cell	Si-cantilevers	Day 14	Electrical	5 V<br>0.5 Hz<br>250 ms	3.52 nN/µm² (Healthy)[g]<br>0.88 nN/µm² (SOD1 E100G)[g]<br>1.05 nN/µm² (SOD1 L144P)[g]	—	—	*Badu-Mensah et al., 2020*
	Human induced pluripotent stem cell	Collagen I-coated cantilever	Day 10 - 11	Electrical	5 V<br>0.5 Hz<br>250 ms	119.8 ± 16 nN<br>(2.94 ± 0.31 V)	—	—	*X. Guo et al., 2020*
	C2C12 myoblasts<br>Chick myoblast<br>Primary Human myoblast	Muscular thin film	3 weeks	Electrical	2 Hz	200 Pa (C2C12)[g]<br>15.625 kPa (Chick)[g]<br>200 Pa (Human)[g]	20 Hz	100 Pa (C2C12)[g]<br>50 kPa (Chick)[g]<br>200 Pa (Human)[g]	*Santoso et al., 2021*
	Human induced pluripotent stem cell	Muscular thin film<br>Elastomeric gelatin substrates	Day 7 - 10	Electrical	20 - 30 V<br>2 Hz	0.986 KPa	20 - 30 V<br>99 Hz	1.986 KPa	*Tanoury et al., 2021*

**POST DEFLECTION**	Mouse myoblasts	Flexible PDMS µPost<br>Collagen Type I and Matrigel<br>Fibrinogen and thrombin	Day 5 - 12	Electrical	—	—	40 V<br>40 Hz<br>4 ms<br>For 1-2 s	10 µN (Day 3)[g]<br>42.5 µN (Day 12)[g]	*H. Vandenburgh et al., 2008*
	C2C12 myoblasts (mouse)	Elastic PDMS cantilevers with fluorescent microbeads<br>Collagen Type I and Matrigel<br>Spring constant (K) = 0.33 mN/mm	Day 14	Optical	High-power LED unit or 300 W mercury lamp (GFP filter)	2.1 µN	—	—	*Sakar et al., 2012*
	C2C12 myoblasts (mouse)	Flexible PDMS 500 µm-µPost<br>Fibrinogen, thrombin and matrigel	Day 3 - 8	Electrical	—	—	20 V<br>30 Hz<br>2 ms<br>For 5 s	6.6 ± 2.2 µN (Day 3)<br>57.5 ± 5.8 µN (Day 6)<br>77.5 ± 11.1 µN (Day 8)	*Shimizu et al., 2017*
	Primary human myoblasts	Square PDMS post in 96 well plate<br>Collagen Type I and Matrigel	Day 14	Electrical	1, 2, 5 Hz<br>5 ms<br>20 mA	75.26 µN (CTRL)<br>79.44 µN (STIM)	20 Hz<br>5 ms<br>20 mA	336.585 µN (CTRL)<br>428.571 µN (STIM)	*Mills et al., 2019*
	Derived-Myoblasts from Human Dermal Fibroblast	PDMS microdevice with flexible µPost<br>Fibrinogen, thrombin and matrigel	Day 4 - 10	Electrical	1 Hz<br>For 5 s	—	30 Hz<br>For 5 s	1.6 ± 1.8 µN (Day 4)<br>4.7 ± 3.4 µN (Day 6)<br>9.7 ± 4.6 µN (Day 8)<br>12.2 ± 5.3 µN (Day 10)	*Shimizu et al., 2020*
	Primary Human Myoblast	PDMS device<br>MyoTACTIC<br>Fibrinogen, thrombin and Geltrex	Day 7 - 14	Electrical	5 V<br>0.5 Hz<br>20% Duty Cycle	—	5 V<br>20 Hz<br>20% Duty Cycle	38 µN (Day 7)<br>122 µN (Day 10)<br>192 µN (Day 14)	*Afshar et al., 2020*
	Immortalized human myoblast	Flexible PDMS 500µm- µPost<br>Fibrinogen, thrombin and matrigel	Day 2 - 8	Electrical	—	—	1.1 V/mm<br>2 ms<br>30 Hz<br>For 5 s	11.3 ± 6.7 µN (Day 4)<br>28 ± 8.4 µN (Day 6)<br>28.5 ± 10.5 µN (Day 8)	*Nagashima et al., 2020*
	Immortalized human myoblast	Flexible PMMA µPost<br>Fibrinogen, thrombin and Geltrex<br>Spring constant (K) = 39.24 ± 0.78 µN/µm	Day 7-14	Optical<br>Biochemical	Optogenetically induced Channelrhodopsin-2	0.2 ± 0.04 mN	2 mM ACh	1.1 ± 0.3 mN	*Hofemeier et al., 2021*
	Immortalized human myoblast	PDMS device<br>MyoTACTIC<br>Fibrinogen, thrombin and Geltrex	Day 10	Electrical	5 V<br>0.5 Hz<br>20% Duty cycle	118.01 µN[g]	5 V<br>20 Hz<br>20% Duty cycle	201.887 µN[g]	*Ebrahimi eet al., 2021*

	Rat myoblasts<br>(adult)	Laminin coated-SYLGARD substrates	Day 31 ± 4	Electrical	70 V<br>4 ms	215 ± 26 μN	40 V<br>40 Hz<br>1.20 ms<br>For 2s	440 ± 45 μN	*Dennis & Kosnik, 2000*
	Rat myoblast<br>(Extensor digitorum longus)	Laminin coated-SYLGARD substrates	Day 32 ± 4	Electrical	0.54 ± 0.08 V/mm<br>4-6 ms	162 ± 13 μN	0.54 ± 0.08 V/mm<br>4-6 ms	281 ± 218 μN	*Dennis et al., 2001*
	Rat myoblasts	SYLGARD Substrates<br>Fibrinogen and thrombin	3 weeks	Electrical	15 V<br>1.2 ms	329 ± 26.3	15 V<br>150 Hz<br>1.2 ms<br>For 1 s	805.8 ± 55	*Huang et al., 2005*
	Rat myoblasts	Laminin coated-SYLGARD substrates	Day 16 - 18	Electrical	2.5, 5, 10 and 20 V<br>1.2 ms	102 μN*	10 V<br>10. 20, 40, 60, 80 Hz<br>1.2 ms<br>For 1 s	212 μN*	*Larkin et al., 2006*
	C2C12 myoblasts (mouse)	Laminin coated-SYLGARD substrates	Day 12 - 16*	Electrical	1 V/mm<br>1 Hz<br>10 ms	0.73 ± 0.42 mN/mm^2	1 V/mm<br>50 Hz<br>10 ms<br>For 2 s	0.88 ± 0.48 mN/mm^2	*Fujita et al., 2009*
**FORCE TRANSDUCERS**	C2C12 myoblasts (mouse)	Polycarbonate cylinder with neodymiun magnet<br>Collagen type I and matrigel	Day 2 - 17	Electrical	15 V<br>10 ms	33.2 μN	15 V<br>10 ms<br>50 Hz<br>For 2 s	—	*Yamamoto et al., 2011*
	Rat myoblasts (neonatal)	25mm silicone tube<br>Fibrinogen, thrombin and matrigel	Day 14	Electrical	3 V/mm<br>10 ms	0.59 ± 0.28 mN (Collagen I)<br>1.68 ± 0.32mN (40% Matrigel)	3 V/mm<br>40 Hz<br>For 1 s	0,88 ± 0.42mN (Collagen I)<br>2.84 ± 0.50mN (40% Matrigel)	*Hinds et al., 2011*
	C2C12 myoblasts (mouse)	Polycarbonate cylinder with neodymiun magnet<br>Collagen type I and matrigel	Day 7	Electrical	20 V<br>10 ms	18.3 ± 2.4 μN	20V<br>10ms<br>50 Hz<br>For 2 s	34.5 ± 2.8 μN	*Sato et al., 2013*
	Rat myoblasts	PDMS molds with semi-cylindrical wells<br>Fibrinogen, thrombin and matrigel	Week 2	Electrical	3 V/mm<br>10 ms	17.83 ± 1mN	3 V/mm<br>10 ms<br>40 Hz<br>For 1 s<br>110 % strecth	28.80 ± 0.93 mN<br>8.25 ± 0.99 μN (Fiber)	*Juhas & Bursac, 2014*
	Primary Human myoblast	PDMS molds within CEREX frame<br>Fibrinogen, thrombin and matrigel	Week 1 - 4	Electrical	40 V/cm<br>1 Hz<br>10 ms<br>2% Stretch	0.403 mN (Week 1)*<br>0.525 mN (Week 2)*<br>0.547 mN (Week 3)*<br>0.701 mN (Week 4)*	40 V/cm<br>5-10-20 Hz<br>For 1 s<br>12% Stretch	0.809 mN (Week 1)*<br>0.986 mN (Week 2)*<br>0.924 mN (Week 3)*<br>1.460 mN (Week 4)*	*Madden et al., 2015*
	C2C12 myoblasts (mouse)	Polycarbonate cylinder with neodymiun magnet<br>Collagen type I	Day 7	Electrical	18 V<br>10 ms	81.264 μN*	18 V<br>10 ms<br>50 Hz<br>For 2 s	151.372 μN*	*Ikeda et al., 2016*

	C2C12 myoblasts (mouse)	Artificial tendons from porcine aorta in a silicone sheet Collagen type I	3 weeks	Electrical	1 V/mm 0.5 Hz 2ms	49.4 ± 8.2 µN (Week 1) 101.3 ± 36.3 µN (Week 2) 166.3 ± 59.4 µN (Week 3)	—	—	*Nakamura et al., 2017*
	hPSC derived human myoblasts	PDMS molds within CEREX frame Fibrinogen, thrombin and matrigel	Week 1 - 4	Electrical	40 V/cm 10 ms 5% Stretch	0.077 mN (Week 1)[g] 0.14 mN (Week 2)[g] 0.176 mN (Week 4)[g]	5-10-20-40 Hz For 1 s 20% Stretch	0.252 mN (Week 1)[g] 0.402 mN (Week 2)[g] 0.427 mN (Week 4)[g]	*Rao et al., 2018*
	C2C12 myoblasts (mouse) Human derived myoblast	3D printed mold Collagen Type I and Matrigel in 50 µL molds	14 Days	Electrical	3.6 V/mmm 1 Hz 1.2 ms	4.87 µN (65% Collagen) 4.16 µN (85% Collagen) (Matrigel/collagen) 48.39 ± 3.49 µN (C2C12) 36 µN[g] (Human)	3.6 V/mmm 100 Hz For 1 s	6.02 µN (65% Collagen) 5.98 µN (85% Collagen) (Matrigel/collagen) 47.74 ± 0.31 µN (C2C12) 47 µN[g] (Human)	*Capel et al., 2019*
	Primary Human Myoblast	PDMS mold Fibrinogen, thrombin and matrigel	Week 1 - 2	Electrical	40 V/cm 10 ms 2% Stretch	0.5 ± 0.03 mN (CTRL) 1.7 ± 0.13 Mn (1 Hz) 1.5 ± 0.16 mN (10 Hz)	40 V/cm 1-5-10-20 Hz 12% Stretch For 1 s	0.9 ± 0.07 mN (CTRL) 3.4 ± 0.18 mN (1 Hz) 3.2 ± 0.19 mN (10 Hz)	*Khodabukus et al., 2019*
	hPSC derived human myoblasts	PDMS molds Fibrinogen, thrombin and matrigel	Week 4	Electrical	24 V 0.5 Hz For 6 s	0.14 ± 0.04 mN (KOSR) 0.811 ± 0.11 mN (KOSR+EGM) 1.393 ± 0.342 mN (KOSR+EGM-to-KOSR)	24 V 20 Hz For 6 s	0.432 ± 0.098 mN (KOSR) 2.0373 ± 0.1315 mN (KOSR+EGM) 2.924 ± 0.517 mN (KOSR+EGM-to-KOSR)	*Xu et al., 2019*
	C2C12 myoblasts (mouse)	Chamber with PTFE Pillars Collagen Type I and Matrigel	10 Days	Electrical	0.83 V/mm 10 ms	1.36 ± 0.21 mN	0.83 V/mm 10 ms For 2 s	1.93 ± 0.12 mN	*Akiyama et al., 2021*
	Primary Human Myoblast	3D Bioprinted dumbbell-shape 4 layer cell/Matrigel molds	Day 17 -19	Electrical	—	—	400 mA 25 Hz 1 ms For 300 ms	175 µN	*Alave Reyes-Furrer et al., 2021*
